# Identification of GOLDEN2-like transcription factor genes in soybeans and their role in regulating plant development and metal ion stresses

**DOI:** 10.3389/fpls.2022.1052659

**Published:** 2022-11-11

**Authors:** Intikhab Alam, Hakim Manghwar, Hanyin Zhang, Qianxia Yu, Liangfa Ge

**Affiliations:** ^1^Department of Grassland Science, College of Forestry and Landscape Architecture, South China Agricultural University (SCAU), Guangzhou, Guangdong, China; ^2^College of Life Sciences, South China Agricultural University (SCAU), Guangzhou, Guangdong, China; ^3^Guangdong Subcenter of the National Center for Soybean Improvement, South China Agricultural University (SCAU), Guangzhou, Guangdong, China; ^4^State Key Laboratory for Conservation and Utilization of Subtropical Agro-Bioresources, College of Forestry and Landscape Architecture, South China Agricultural University (SCAU), Guangzhou, Guangdong, China

**Keywords:** soybean, Golden2-like TF, phylogenetic classification, duplication, gene expression, metal ion stress

## Abstract

The Golden 2-Like (G2-like or GLK) transcription factors are essential for plant growth, development, and many stress responses as well as heavy metal stress. However, G2-like regulatory genes have not been studied in soybean. This study identified the genes for 130 G2-Like candidates’ in the genome of *Glycine max* (soybean). These GLK genes were located on all 20 chromosomes, and several of them were segmentally duplicated. Most GLK family proteins are highly conserved in Arabidopsis and soybean and were classified into five major groups based on phylogenetic analysis. These *GmGLK* gene promoters share cis-acting elements involved in plant responses to abscisic acid, methyl jasmonate, auxin signaling, low temperature, and biotic and abiotic stresses. RNA-seq expression data revealed that the GLK genes were classified into 12 major groups and differentially expressed in different tissues or organs. The co-expression network complex revealed that the *GmGLK* genes encode proteins involved in the interaction of genes related to chlorophyll biosynthesis, circadian rhythms, and flowering regulation. Real-time quantitative PCR analysis confirmed the expression profiles of eight GLK genes in response to cadmium (Cd) and copper (Cu) stress, with some GLK genes significantly induced by both Cd and Cu stress treatments, implying a functional role in defense responsiveness. Thus, we present a comprehensive perspective of the GLK genes in soybean and emphasize their important role in crop development and metal ion stresses.

## Introduction

Soybean is a significant oil and food crop with a rich protein content and sources of food and cooking oil worldwide (Kim et al., 2017). However, soybean crops are frequently exposed to a variety of environmental stresses, which restrict crop yields. Accumulation of heavy metals in soil and water can be attributed to several anthropogenic activities such as industrialization and modern farming practices, including extensive exploitation of land resources (Salazar et al., 2012; Zhang et al., 2019). Heavy metal buildup in plant tissues may hinder the plant’s significant enzymatic activity, causing a variety of negative effects on germinability, seedling growth, and photosynthetic activity (Sharma and Dietz, 2006). Heavy metals are taken up by the roots of plants and moved to the shoots. This has a negative effect on root and shoot cells as well as organelles such as chloroplasts and mitochondria, which limits energy production and enforces peroxidation (Garg and Singla, 2011; Yan et al., 2020).

Plants have evolved complex defense systems to counteract the effects of environmental factors (Bohnert et al., 2006). Transcription factors (TFs) are important regulators of developmental processes and stress responses, playing a crucial role in signal transduction and gene expression regulation (Davidson et al., 1983; Suo et al., 2003; Ramsay and Glover, 2005; Shin et al., 2016; Zaikina et al., 2019). These TFs are altered during stress, which affects their intracellular allocation, consistency, activity, connections with several other proteins, and eventually the expression of target genes. MYB-like TFs are among the most significant TF families engaged in the plant transcriptional control network and are linked to many stressors, including metal ion stress (Hu et al., 2017; Jalmi et al., 2018; Li X. et al., 2021; Wa Lwalaba et al., 2021). Currently, the completion of whole genome sequencing has allowed us to comprehensively analyze and classify the TFs; however, several of them remain unknown.

Golden2-Like (G2-Like or GLK) transcription factors occur widely in plants. They belong to the GARP superfamily in the Myb class of transcription factors. They play important roles in chlorophyll biosynthesis, leaf senescence, and stress responses, including heavy metal stress (Riechmann et al., 2000; Fitter et al., 2002; Van De Mortel et al., 2008; Kakizaki et al., 2009; Waters et al., 2009; Kobayashi et al., 2013; Garapati et al., 2015; Leister and Kleine, 2016; Nagatoshi et al., 2016; Ahmad et al., 2019). The first GLK transcription factor was reported in *Zea mays* (Hall et al., 1998). A typical GLK protein contains two conserved domains: a Myb-DNA binding domain (DBD) and a GCT box (Rossini et al., 2001). In Arabidopsis, *AtGLK1* and *AtGLK2* have been shown to regulate chloroplast development (Fitter et al., 2002; Yasumura et al., 2005; Waters et al., 2008). Furthermore, *AtGLK2* is important for anthocyanin production. In Arabidopsis seedlings, the accumulation of anthocyanins is limited due to loss of function of *AtGLK2*, and its overexpression increased the accumulation of anthocyanins (Liu et al., 2021a). In barley, the G2-like ALM1 mutant reduces seed weight (Taketa et al., 2021). The overexpression of two *ZmGLK* genes, particularly that of the *ZmGLK2* gene governed by the maize UB promoter has recently shown to increase rice yield by 30-40% (Li et al., 2020). The overexpression of *AtGLK1* gene under control of its silique promoter (PAt1G56100) increased 11% seed weight in Arabidopsis. Moreover, overexpressing *AtGLK1* with its leaf promoter increased leaf photosynthesis and 25% of seed yield (Zhu et al., 2018). Additionally, it has been demonstrated that GLK proteins act with ANAC92 to exhibit leaf senescence (Rauf et al., 2013). Arabidopsis atglk1/atglk2 double mutants exhibited leaf senescence in addition to plant yellowing, whereas plants overexpressing *AtGLK1* or *AtGLK2* exhibited the opposite phenotype (Waters et al., 2009). In atglk1/atglk2 double mutants, overexpression of *AtGLK1* or *AtGLK2* can complement their progeria phenotypes (Waters et al., 2008). GLK genes are also involved in plant senescence and are regulated by signaling pathways such as light, ABA, and brassinolide (BR). Light is required for chloroplast development but is involved in plant senescence by inducing the expression of AtGLKs genes (Fitter et al., 2002). In addition, *AtGLK1* modulates the expression of disease-resistance genes and has varied effects on diverse pathogens (Chen et al., 2016). *AtGLK1* increases resistance to *Fusarium graminearum* in Arabidopsis and promotes cucumber mosaic virus tolerance (Savitch et al., 2007; Schreiber et al., 2011; Han et al., 2016). *AhGLK1b* can enhance tolerance to fungal and bacterial infections and other environmental stresses (Liu et al., 2021b). In rice, *OsGLK1* is involved in disease resistance (Chen et al., 2016). Loss of function of *SlGLK29* may affect cold tolerance in tomato plants (Junfang et al., 2017). Similarly, *GhGLK1* in cotton has been associated with the response to cold and drought stress (Liu et al., 2021b). Several GLK gene functions have been studied extensively. Nevertheless, those associated with abiotic stress have received little attention, and only a few published scientific studies are available. Previously, GLK family genes were discovered and studied in the genomes of several plant species, including Arabidopsis (Alam et al., 2022), cotton (Zhao et al., 2021), maize (Liu et al., 2016), tomato (Wang et al., 2022), and tobacco (Qin et al., 2021). However, there have been no report on the GLK gene family in the soybean genome.

This study identified and classified soybean GLK gene-containing proteins based on phylogenetic tree analysis. Furthermore, the *GmGLK* genes were analyzed and annotated. The cis-regulatory elements of the *GmGLK* promoter region were examined. Interestingly, several of these GLK genes have been shown to be expressed in various soybean tissues. Furthermore, the expression analysis of eight GLK genes in response to cadmium and copper treatments were confirmed using real-time quantitative PCR (qRT-PCR). Our research will not only expand genetic research on GLK genes in soybean while also give a valuable perspective and new insights for researchers to investigate *GmGLK* functions in the future.

## Materials and methods

### Identification and analysis of soybeans GLK members

The entire set of previously reported genomic and proteomic data on Arabidopsis GLK genes was downloaded from the Arabidopsis database TAIR (http://www.arabidopsis.org) and used as query sequences for BLASTp searches against Phytozome V13 plant databases. To confirm the existence of GLK-associated motifs, all GLK sequences were screened using Hidden-Markov-Model (HMM) profiles and online tool such as SMART (http://smart.embl-hei-delberg.de), Pfam (http://pfam.sanger.ac.uk), and Interpro (http://www.ebi.ac.uk/interpro/). The ProtParam tool was searched to examine the isoelectric point (pI) and molecular weight (Wt) (Protparam, 2017). Furthermore, the PlantRegMap tool was explored to analyze the gene ontology (GO) of the soybean GLK genes.

### Phylogenetic tree analysis

Phylogenetic analysis of Arabidopsis and soybean GLK proteins was generated using MEGAX software with the maximum likelihood (ML) algorithm (Kumar et al., 2018) and Jones-Taylor-Thornton (JTT). Bootstrap with 1000 replications was used to evaluate the group support (Jones et al., 1992).

### Structure of GmGLK genes, conserved motifs, and phylogenetic analysis

TBtools was used to depict the exon-intron arrangement of the GLK-encoding genes (Chen et al., 2020), the MEME tool for search conserved motifs in GLKs (Bailey and Elkan, 1994), and Tomtom was used to predict the TFs that were most likely to bind to these predicted binding sites (Gupta et al., 2007).

### Analysis of GmGLK promoters, expression pattern, and co-expression analysis

Phytozome V13 plant databases (https://phytozome-next.jgi.doe.gov/) were searched to retrieve the promoter sequences (2-kb upstream genomic region) of the *GmGLK* genes. The obtained upstream regions were submitted to the online Plant-CARE web tools for important cis-elements of the GLK genes (Lescot et al., 2002). The soybean GLK gene expression data files (RNA-seq expression data) were obtained and examined from the Phytozome V12.1 database. The *GmGLK* protein list was obtained from the “CoExSearch” tool and submitted to the STRING web tool to predict putative interacting proteins (Szklarczyk et al., 2018). An interaction network was generated through the Cytoscape tool.

### Physical location of GmGLKs on the chromosome and Ka/Ks analysis

The chromosomal location of soybean GLK genes was determined using genome annotation files. The allocation and segmental duplication of soybean GLK genes on chromosomes were mapped and depicted using MapMan tools. The duplicated *GmGLK* genes were shown *via* numerous color lines. Clustal Omega was used for sequence alignments (Sievers and Higgins, 2014), and the ratios of synonymous (Ks), non-synonymous (Ka), and evolutionary pressures (Ka/Ks) among the *GmGLK* pairs were estimated *via* the PAL2NAL and PAML package (Goldman and Yang, 1994; Yang et al., 1994; Suyama et al., 2006).

### Plant materials and qRT-PCR analysis

Good quality Williams 82 cultivar Soybean seeds were apparent clean with 1% sodium hypochlorite for five min with gentle shaking before being washed with ddH_2_O. Then the clean seeds were sown in a mixture of soil and sand-filled pots (1:1) and cultivated in a control chamber with 16/8- h of the light–dark cycle at 22°C and 65-70% humidity. To examine the expected role of *GmGLK* genes in response to metal stress treatment, seven days of soybean seedlings were exposed to an excess amount of Cd (50 µM of CdCl_2_) and Cu (50 µM of CuSO_4_·5H_2_O) for 1–6 hour treatments. Total RNA was isolated from the frozen roots of each soybean (0.3 cm) utilizing a plant-specific RNA extraction kit according to the company’s guidelines (OMEGA, China). For each treatment, three biological replicates were prepared to reduce the error rate. The RNA quality was evaluated using electrophoresis and the NanoDrop 2000 Spectrophotometer (Thermo Fisher Scientific, USA). To generate first-strand cDNA from 1 ug of total RNA from each sample, the cDNA Synthesis Kit (Takara, China) was used. Before use, the reverse transcription products were diluted 20 times and stored at 20°C. The online tool Primer3Plus was employed to construct GLK genes specific primers in soybean. Soybean actin primers were used as a control (**Table S6**). Quantitative RT-PCR was performed using a CFX-Bio-Rad RT System. The 2−ΔΔCt method was used to evaluate the data. The relative expression levels were normalized to those of the housekeeping genes.

## Results

### Identification of GmGLK genes containing protein subsection

To explore the *GmGLK* genes containing proteins in soybean, we accomplished a BLASTp search in the Phytozome v13 databases utilizing previously available *AtGLK* proteins from Arabidopsis (Alam et al., 2022). The non-redundant soybean GLK proteins were screened through SMART and Pfam online tools for the existence of a Myb-like domain. In total, 130 GLK genes were detected in the soybean genome, and were named *GmGLK1* to *GmGLK130* according to the location on the chromosome. Detailed information about *GmGLK* genes, including gene ID, genomic location, length of gene/protein, isoelectric point (pI), and molecular weight (Wt) is listed in **Table S1**. Furthermore, each of the identified *GmGLK* proteins was screened for the existence of any other domain in addition to the GLK domain (s). Six additional domains were identified, allowing the organization of 130 *GmGLK* proteins into seven groups (**Table S2**). Group I contained 60 *GmGLK* proteins (46.15%). None of these members had additional domains apart from the GLK domain. Group II comprised of 35 *GmGLK* members (26.92%) with an additional Myb-CC_LHEQLE domain. Group III included 27 GLK members (20.77%) with an additional REC domain, Group IV included two *GmGLK* proteins (1.54%) with an additional coiled-coil domain, and the remaining groups contained one member each with one or more additional domains (**Table S2**). GO analysis demonstrated that all soybean GLK proteins had DNA-binding activity, were primarily found in the nucleus, and were involved in a variety of biological activities in the cell (**Table S3**).

### Evolutionary relationship of GLK genes containing proteins

**Figure 1:**
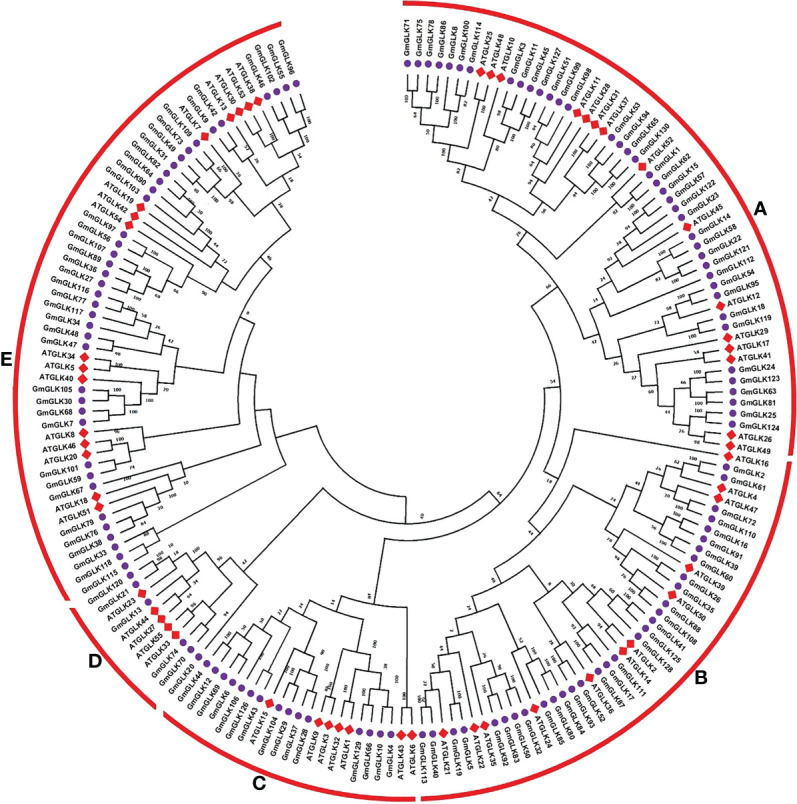
Phylogenetic tree and classification of *Glycine max* and *A. thaliana* GLK proteins. The ML-hood with Jones Taylor Thornton (JTT) model tree was constructed using MEGAX. The bootstrap support from 1000 replications is transformed to each node. The GLK proteins were clustered into five major groups **(A–E)**.

A phylogenetic tree was generated based on Arabidopsis and soybean GLK genes encoding proteins through available MEGAX software with the option of the maximum likehood (ML) technique (**Figure 1**). Based on these results, all GLK members were clustered into five major groups (Groups A-E), with Arabidopsis and soybean orthologous or homologous proteins clustered together (**Figure 1**). Group A contained 54 (15 *AtGLK*, 39 *GmGLK*), group B contained 43 (12 *AtGLK*, 31 *GmGLK*), group C contained 22 (7 *AtGLK*, 15 *GmGLK*), group D contained 11 (5 *AtGLK*, 6 *GmGLK*), and group E contained 55 (16 *AtGLK*, 39 *GmGLK*) proteins (**Figure 1**).

### Analysis of soybean GLK gene structure and conserved motif composition

**Figure 2:**
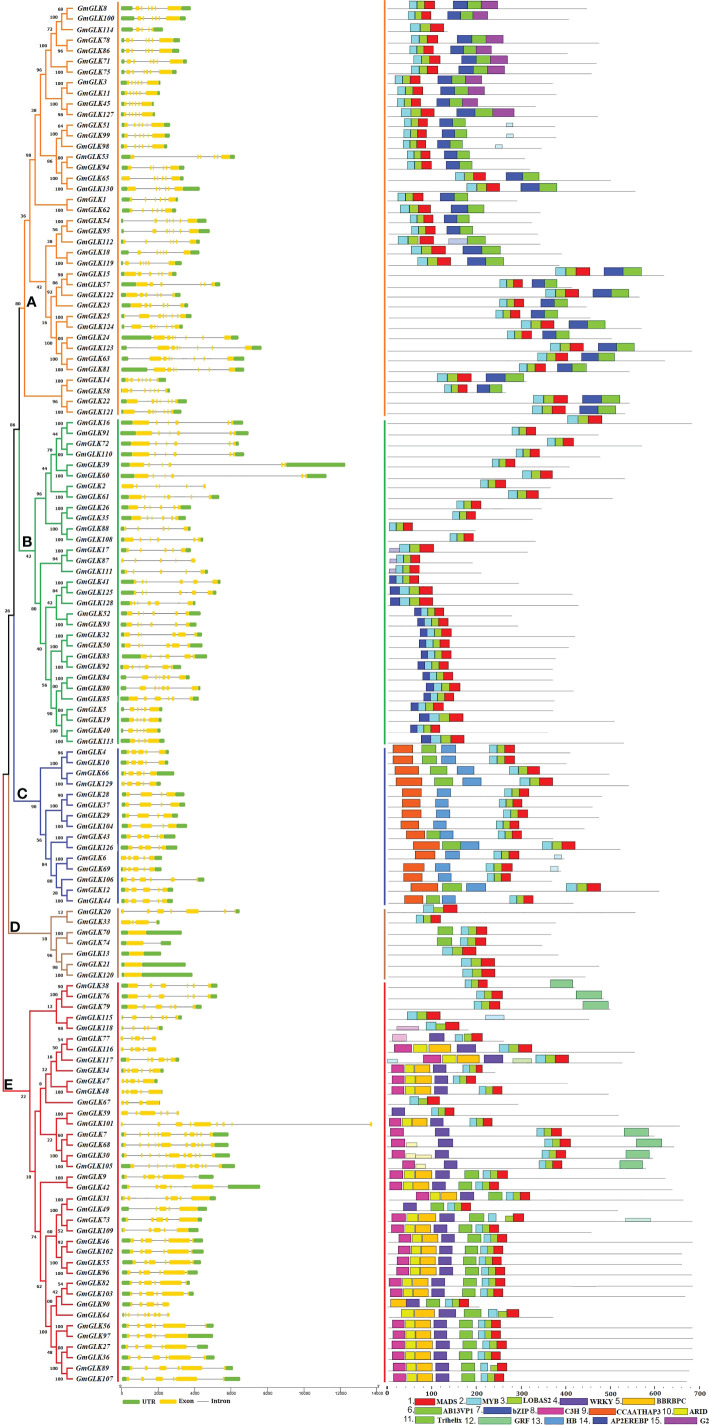
Schematic representation of the structural organization and motif composition of the soybean GLK genes. The ML approach evolutionary tree is presented on the left, which was clustered into five major groups **(A–E)**, preceded by the GLK gene structure, including exons and introns, which are represented by yellow color boxes and black lines, respectively. The preserved motifs are shown in different colors. Non-conserved regions are shown in black lines.

The generated phylogenetic tree was analyzed for evolutionary relationships between GLK members using 130 *GmGLK* protein sequences (**Figure 2**). The numbers of exons and introns from one to eleven in soybean GLK genes. The gene structures were generally well-preserved within the variant groups of the phylogenetic tree classification. However, divergence in exon and intron numbers and lengths was observed between each group.

To study the structural variations of the soybean GLK proteins, we observed the conserved motifs organization of each GLK protein from soybean according to phylogenetic classification (**Figure 1**). As shown in **Figure 2** and **Figure S1**, 15 conserved motifs were identified using MEME software with a range of 15 - 50 amino acids, and Motifs 1-3 were distributed across all GLK members (which contain the MADS, MYB, and LOBAS2 signature motifs). In addition, the remaining motifs were distributed among the different groups of the phylogenetic tree classification and could conceivably be used to distinguish between subfamilies. Group A includes motifs 6, 7, and 15 (which mostly contain Ab13vp1, bZIP, and G2-like signature motifs). Group B includes motif 14 (which contains the AP2EREBP signature motif). Group C includes motifs 9 and 13, whereas some members have motif 11 (which contains the CCAATHAP3, HB, and Trihelix signature motifs). Group D contains only two members with an additional motif 11 (which contains the Trihelix signature motif), and group E includes motifs 4, 5, 10, 11, and 12 (which contain the WRKY, BBRBPC, ARID, Trihelix, and GRF signature motifs) (**Figure S1**).

### Physical location of *GmGLKs* on the chromosome and Ka, Ks analysis

**Figure 3:**
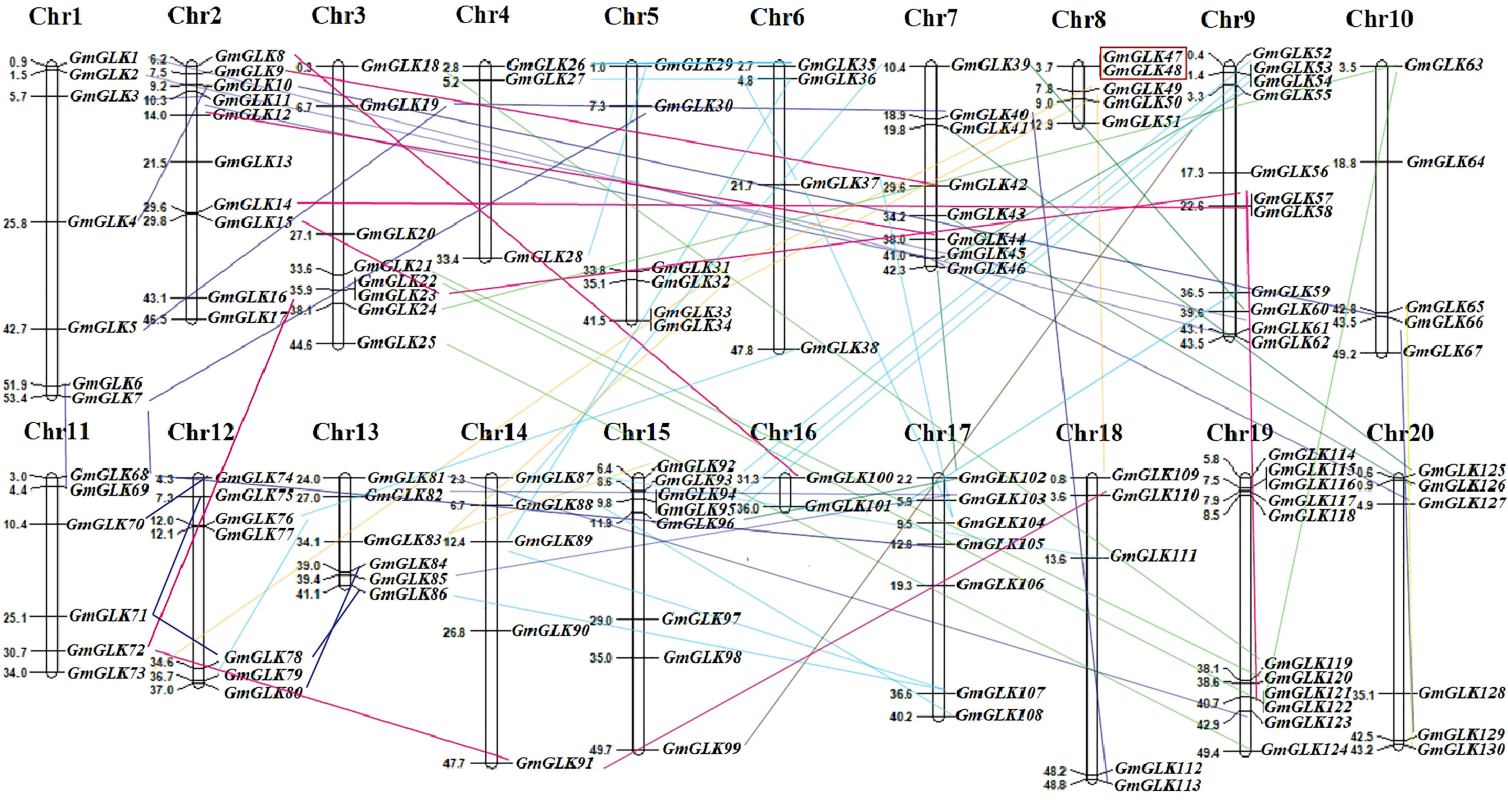
The distribution of GmGLK members on 20 soybean chromosomes.The chromosome number (Chr1-Chr20) and the name and physical position (Mb) of GLK members are represented on each chromosome. The segmentally duplicated members are connected with the variant lines, whereas the tandem duplicated members are shown in red color box.

The chromosomal location of each soybean GLK member was retrieved from the Phytozome v13 database (**Table S1**) and mapped onto specific soybean chromosomes (**Figure 3**). The 130 *GmGLK* genes were positioned on 20 chromosomes, including seven *GmGLK* genes on Chr-1, ten on Chr-2, eight on Chr-3, three on Chr-4, six on Chr-5, four on Chr-6, eight on Chr-7, five on Chr-8, eleven on Chr-9, five on Chr-10, six on Chr-11, seven on Chr-12, six on Chr-13, five on Chr-14, eight on Chr-15, two on Chr-16, seven on Chr-17, five on Chr-18, eleven on Chr-19, and six on Chr-20 (**Figure 3**). Our mapping analysis revealed that 95 out of 130 (73.07%) *GmGLK* pairs of genes were involved in segmental duplications, and one pair in tandem duplication (**Figure 3**). Gene duplication is a major contributor to the gene family expansion throughout the genome’s expansion (Cannon et al., 2004). The ancient tetraploid soybean has experienced two round of genome duplications (Zhao et al., 2017). Most soybean genes are paralogous, meaning that they exist in many numbers. To examine the evolutionary history of *GmGLK* duplicated members, the Ka, Ks, and Ka/Ks ratios between members of the paralogous pairs were analyzed (**Table S4**). The Ka/Ks ratios ranged from 0.0313–0.7109, with an average of 0.3375 indicating that *GmGLK* genes have undergone strong purifying selection during evolution. The probable divergence times among the segmentally duplicated *GmGLK* gene pairs ranged from 4.598–94.844 million years (my) with an average of 36.195 my (**Table S4**), indicating that these gene pairs in soybean plant underwent duplication events at approximately 11–35 and 110–170 million years ago (mya).

### Promoter analysis of soybean GLK genes

**Figure 4:**
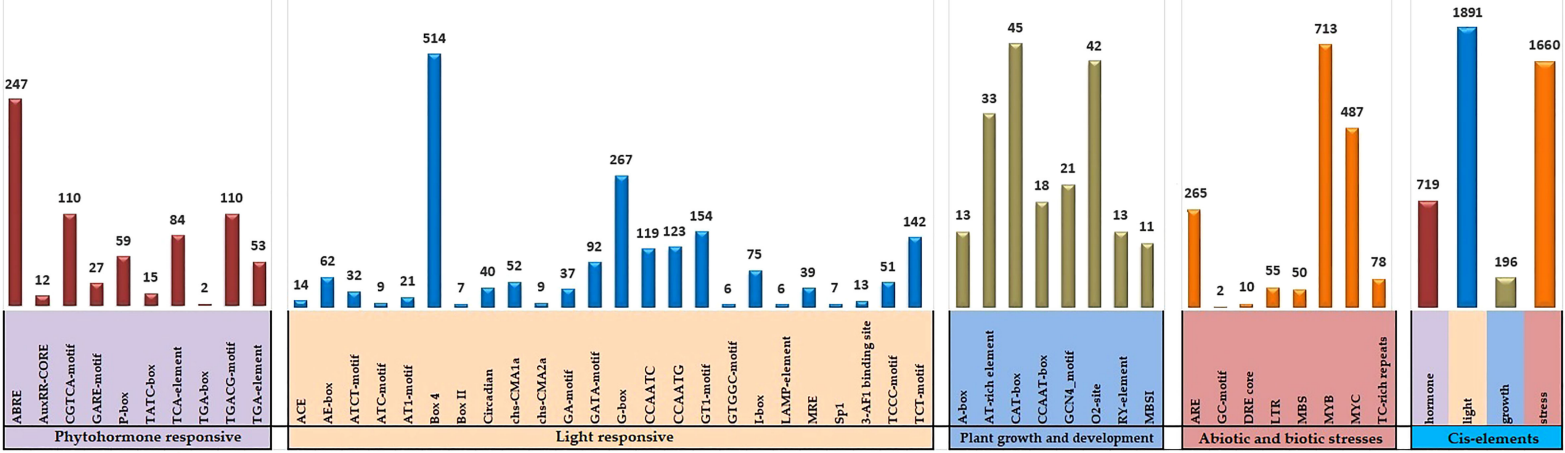
The number of cis-acting elements analyzed in the promoter region of *GmGLK* genes. A separate colored histogram shows the ratio of detected cis-regulatory components of each group.

Cis-acting elements found in the promoter region can assist in determining the activity of candidate genes. The 2-kb segment upstream of the soybean GLK genomic sequences from Phytozome v13 and was submitted to the PlantCARE database. The entire 130 *GmGLK* gene promoter regions with 4466 potential cis-acting elements were discovered. They were divided into four major groups: light-responsive (21), phytohormone responsive (10), plant developmental responses (8), and stress-responsive (8) (**Table S5**). Three light-responsive elements (Box 4-motif, G box-motif, and TCT-motif), three plant hormone-related elements (ABRE, CGTCA-motif, and TGACG-motif) involving ABA, methyl jasmonate, and auxin signaling), three developmental responses (CAT-box motif, O_2_-site motif, AT-rich element), and other stress-responses (ARE-motif, MYB-motif, and MYC-motif) were detected at high ratios in the soybean GLK genes promoter regions (**Figure 4**).

### Expression profiles of GmGLK members across different tissues

**Figure 5:**
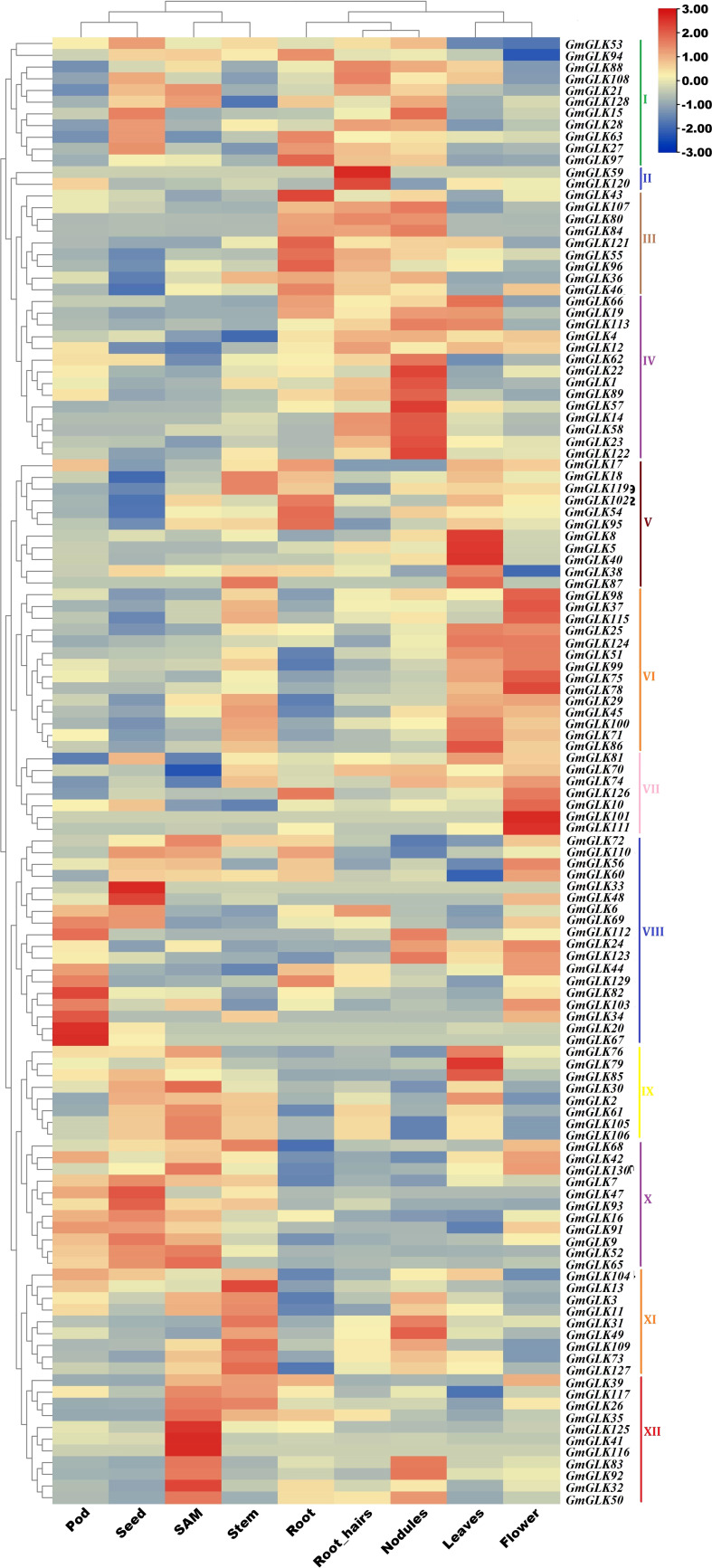
The expression pattern of soybean GLK members in various tissues/organs. The Phytozome database V12.1 was utilized to retrieve the expression data of *GmGLK* genes across numerous tissues. The relative bar level is on the top, and the types of tissue or organ are named on the bottom. The names of the *GmGLK* genes are present on the right side of the heatmap.

Soybean GLK gene expression patterns were obtained by examining their RNA-seq expression files from the Phytozome V12.1 database. The retrieved expression values (FPKM) were log2-transformed, and a clustering heat map illustrating the expression profiles of GLK genes in different tissues or organs was constructed (**Figure 5**)—revealing 125 *GmGLK* genes were clustered into 12 main groups (**Figure 3**). Group I contained 11 *GmGLK* genes, which are mostly expressed in four tissues or organs (seed, root, root hair, and nodules). Group II included two *GmGLK* genes that are specifically and highly expressed in root hair tissue. Group III included nine genes, that are highly expressed in the root, followed by root hair and nodules. Group IV included 14 genes that are specifically and highly expressed in nodules. Group V contained 11 genes. The majority of these genes are highly expressed in the leaves, whereas five are expressed in the roots. Group VI contained 14 genes that are highly expressed in flowers and leaves. Group VII included seven genes that are highly expressed in flowers. Group VIII comprised 18 genes, most of which are highly expressed in pods, followed by seeds and flowers. Group IX included eight genes; that are highly expressed in shoot apical meristem (SAM), followed by seeds and leaves. Group X included 11genes The majority of these genes are highly expressed in seeds, followed by pods and SAMs. Group XI includes 9 genes, which are highly expressed in stem, followed by nodules Group XII includes 11 genes explicitly enriched in SAM.

### Expression of soybean GLK genes in response to cadmium and copper stresses

Heavy metal pollution is regarded as one of the most important societal concerns (Mustafa and Komatsu, 2016). When heavy metal concentrations in soil exceed a certain threshold, plant photosynthesis is inhibited, and nutrient uptake is inadequate, harshly restricting plant production (Cai, 2011; Wang et al., 2020a). Cd has the highest mobility and toxicity compared with that of other heavy metals and is hence one of the most harmful metal contaminants in plants and animals. Cd^+^ affects plant cellular activities, decreases root development, impairs regulatory processes, induces oxidative stress, inhibits nutrient acquisition, damages membranes, and may promote cell death under highly toxic conditions (Song et al., 2017; Chang et al., 2019). Based on our previously study in Arabidopsis, we selected eight orthologous genes in soybean to further understand their expression under Cd and Cu stress in soybean crops (Alam et al., 2022).

**Figure 6:**
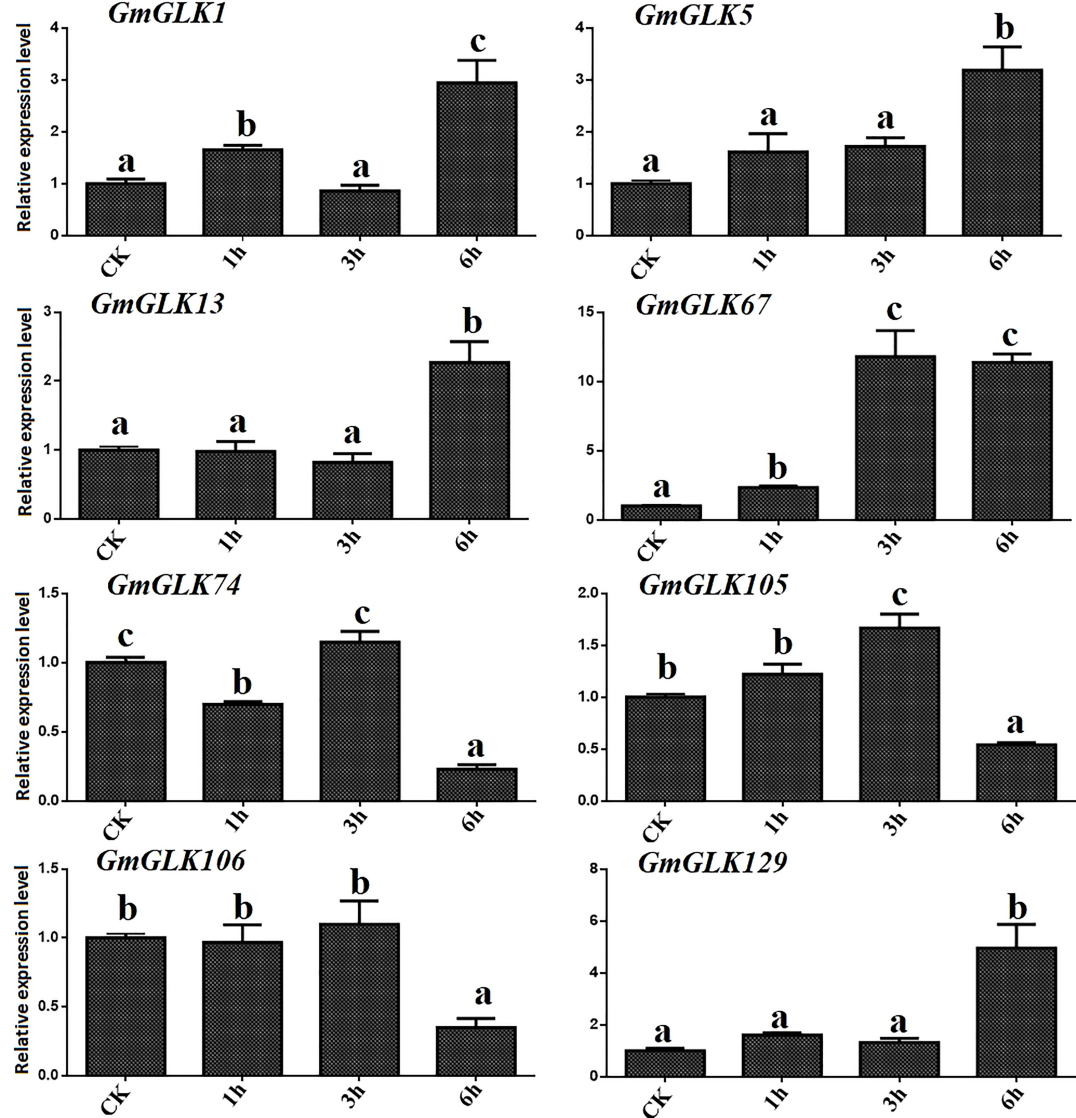
qRT-PCR analysis of the relative expression of GLK members in soybean under Cd stress. The time in hours is represented through the x-axis and relative expression level through y-axis. Tukey’s HSD tests were utilized to measure the differences among effects on various time frames for Cd exposure. The alphabetical letters indicate significant difference (p<0.05).

The relative expression of the eight *GmGLK* members in soybean seedlings exposed to Cd treatment for 1-6 hours was examined using qRT-PCR (**Figure 6**). The relative expression of six *GmGLK* genes was improved by exposure to Cd stress, including five GLK genes, namely *GmGLK1*, *GmGLK5*, *GmGLK13*, *GmGLK67*, and *GmGLK129*, which were 1–12-fold upregulated after exposure of Cd stress for 6 h. In addition, three *GmGLK* members, *GmGLK74*, *GmGLK105*, and *GmGLK106*, were downregulated after 6 h of Cd exposure. However, most *GmGLK* members were downregulated after 1 and 3 h of Cd exposure and upregulated after prolonged exposure to Cd (**Figure 6**).

**Figure 7:**
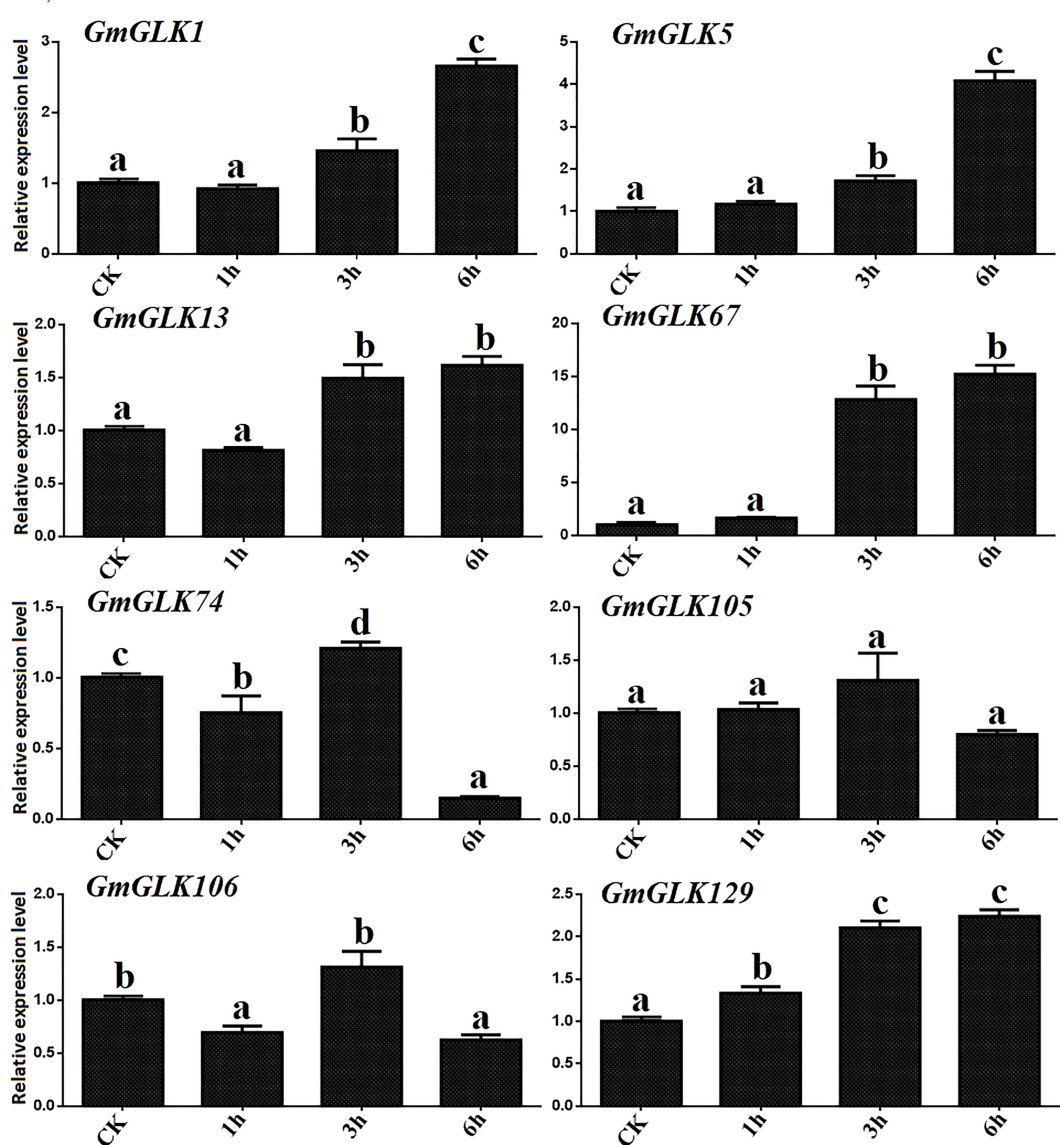
qRT-PCR analysis of the relative expression of GLK members in soybean under Cu stress. The time in hours is represented through the x-axis, and the relative expression level through y-axis. Tukey’s HSD tests were utilized to measure the differences among effects on various time frames for Cu exposure. The alphabetical letters indicate significant difference (p<0.05).

Cu ion concentration significantly influences several metabolic pathways implicated in plant development. Both the excess and deficiency of Cu can seriously affect metabolic activities *in vivo*, such as limiting plant growth (Adrees et al., 2015). In addition, the expression of eight *GmGLK* members was examined using qRT-PCR in soybean seedlings exposed to Cu treatment for 1-6 hours (**Figure 6**). Four *GmGLK* genes—namely *GmGLK1*, *GmGLK5*, *GmGLK67*, and *GmGLK129* were 1 to 14 fold upregulated on exposure to Cu stress,. In contrast, three *GmGLK* genes, including *GmGLK74*, *GmGLK105*, and *GmGLK106* were downregulated following a 6-h exposure to Cu (**Figure 7**).

### Co-expression network analysis of GmGLKs

**Figure 8:**
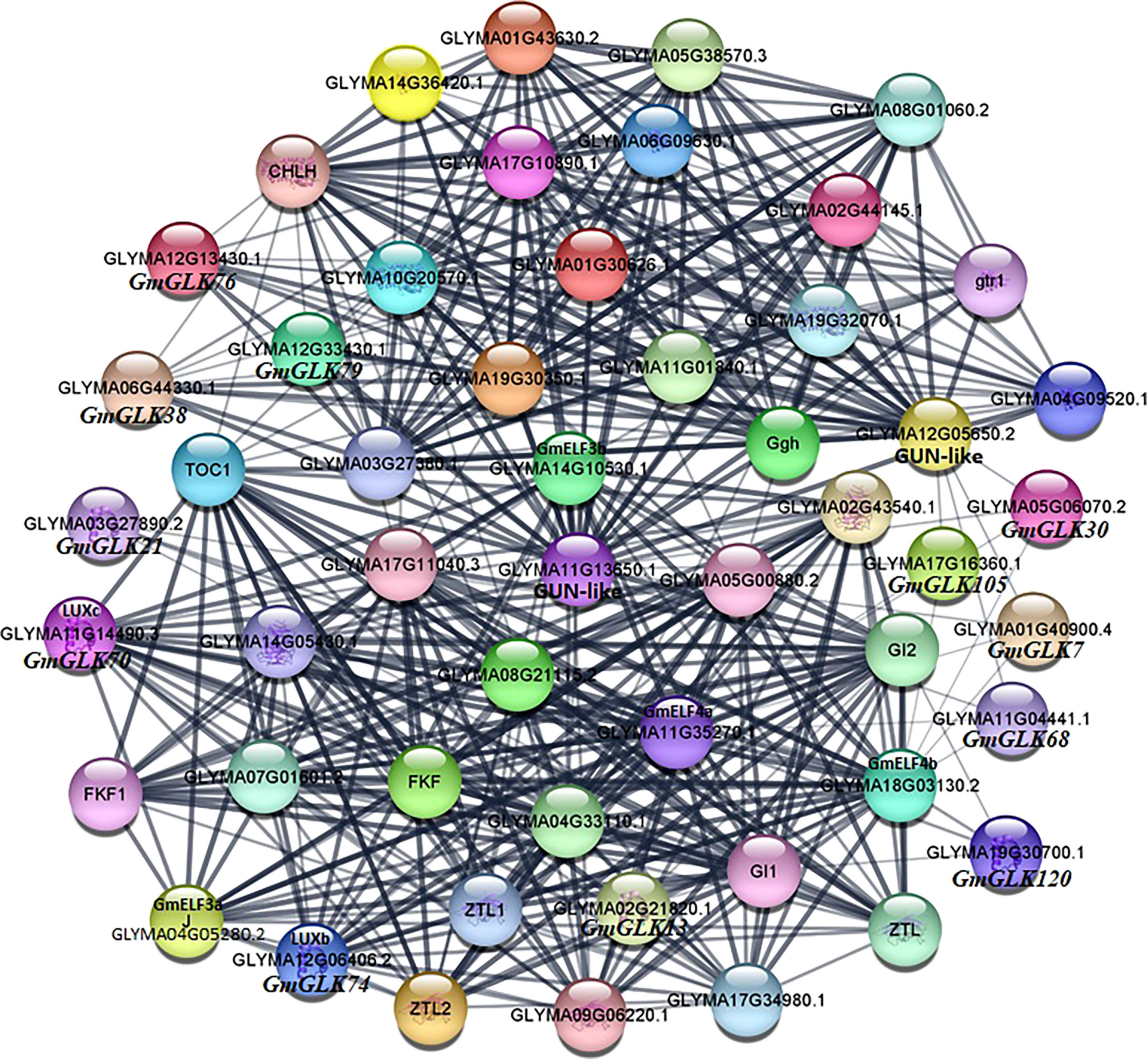
Co-expression networks of *GmGLK* proteins in soybean. The nodes indicate distinct proteins, whereas the edges indicate the interaction across proteins. The width of the edge representing the strength of the interaction.

Different proteins interact with one another to generate a protein interaction network, that contributes to the regulation of signal transmission and gene expression. To further investigate the role of GLK proteins in soybean, we examined the protein-protein interaction network of 130 soybean GLK proteins (**Figure 8**). The results showed that *GmGLK* plays pivotal roles in this network and involved in chlorophyll biosynthesis, circadian rhythms, and flowering regulation. A strong interaction was detected between photosynthesis-related genes and soybean GLK genes, especially *GmGLK38*, *GmGLK76*, and *GmGLK79*—suggesting that these genes may play an essential role in chloroplast development (Nakamura et al., 2009; Kobayashi et al., 2013; Shi et al., 2017). In addition, nine *GmGLK* genes were associated with circadian rhythms, and flowering-related genes—including *GmGLK13*, *GmGLK70* (GmLUXc), *GmGLK74* (GmLUXb), and *GmGLK120*—had strong interaction with *GmELF3a*, *GmELF3b*, *GmELF4b*, and *GmELF4b* genes—also related to the circadian clock and flowering (Nusinow et al., 2011; Preuss et al., 2012; Liew et al., 2017; Uehara et al., 2019).

## Discussion

The G2-Like or GLK transcription factor belongs to the Myb transcription factors of the GARP superfamily. It is present in numerous plant species, including Arabidopsis, cotton, maize, and tobacco, and is involved in several stress responses (Liu et al., 2016; Qin et al., 2021; Zhao et al., 2021; Alam et al., 2022). However, to date, no study has reported on these proteins in soybean species. In the current study, we identified 130 GLK genes in soybean. All GLK protein members in soybean contain a Myb-like domain and various additional domains, including Myb CC LHEQLE, REC, transmembrane, coiled-coil, Hox, and DUF4281 domains. The presence of multiple domains in a protein signifies their evolution of the protein to perform numerous functions.

Analysis of evolutionary relationships shows that the *GmGLK* genes can be classified into five major groups with subgroups. The presence of a substantial bootstrap value on the inner tree branches suggests the presence of homologous proteins with the same activities from a common ancestor. Gene duplications are crucial for neofunctionalization and functional divergence (Birchler and Yang, 2022). Duplications, whether segmental or tandem, are major forces driving gene family expansion (Cannon et al., 2004). In this study, 95 out of 130 (73.07%) pairs of *GmGLK* members were involved in segmental duplications, and one pair in tandem duplication. Previous research has shown that the soybean genome has experienced two rounds of segmental duplication across its evolutionary history, with approximately 75 percent of the genes existing in many copies (Schmutz et al., 2010). The segmentally duplicated pairs diverged between 4.598 and 94.844 my, with an average of 36.195 my (**Table S4**). The results revealed that soybean has undergone two rounds of gene duplication events of about 11–35 and 110–170 mya, and the number of chromosomes in the soybean plant increased from 10 to 20 (Xu et al., 2011). The Ka/Ks ratio of all *GmGLK* duplicated members was reported to range from 0.0313 and 0.7109, and an average of 0.3375, implying the influence of selective pressure on the evolution of these pair of genes because a gene pairs with Ka/Ks less than one could specify purifying selection acting on the diverse protein-encoding genes throughout evolution (Tien et al., 2015).

The phylogenetic tree was generated from 55 *AtGLK* and 130 *GmGLK* genes, which were further distributed into five main groups (A, B, C, D, and E). As indicated in **Figure 1**, all phosphate starvation response (PHR1) genes were clustered into group A, which is the key regulator of the phosphate deprivation response (PDR) in Arabidopsis and rice (Bari et al., 2006; Zhou et al., 2008; Sun et al., 2016). KANADI (KAN)-like genes were found in group B, and they play important roles in organ positioning, cell type patterning, and organ morphogenesis of the SAM (Caggiano et al., 2017; Ram et al., 2020). Members of NIGT1/HRS1/HHO-like genes were clustered in group C. NIGT1 transcription factors have been reported to coordinate N and P responses in Arabidopsis (Kiba et al., 2018; Maeda et al., 2018; Ueda et al., 2020; Wang et al., 2020b; Ludewig et al., 2021). Eleven GLK members were clustered into subgroup D, including AtLUX (*AtGLK33*), AtMYBC1 (*AtGLK23*), and AtBOA (*AtGLK55*), which play important role in circadian oscillation were clustered into subgroup D (Bhutia et al., 2020; Alam et al., 2022). However, 55 GLK members were clustered into subgroup E, including *AtGLK1* and *AtGLK2*, involved in chloroplast formation (Fitter et al., 2002; Yasumura et al., 2005; Nagatoshi et al., 2016), and *AtGLK2*, which also plays a significant role in anthocyanin biosynthesis (Liu et al., 2021a).

The gene architectures were well conserved within a distinct class of phylogenetic categorization, and 15 conserved motifs were found in soybean GLK proteins; a comparable motifs numbers and organization were also reported in Arabidopsis and cotton (Zhao et al., 2021; Alam et al., 2022). The upstream region of the *AtGLK* genes contains essential cis-acting elements associated with phytohormones (ABRE, CGTCA, and TGACG), light response (G-box, Box 4, and TCT), developmental like (AT-rich motif, CAT-box, CCAAT-box), and stress-responsive (ARE, MYB, and MYC), which indicates that underlying hormones and environmental signals can regulate the *GmGLK* gene expression, similar results were reported by other studies in Arabidopsis (Alam et al., 2022), cotton (Zhao et al., 2021) and tobacco (Qin et al., 2021).

Numerous studies have indicated that the *GmGLK* genes are TF that play important roles in plant development (Hall et al., 1998; Fitter et al., 2002; Yasumura et al., 2005). The expression analysis revealed that 125 *GmGLK* genes were classified into 12 groups and were mainly expressed in one or several of the tested tissues or organs, demonstrating the functional diversity of soybean crops. Several members of the *GmGLK* family that exhibit tissue-specific expression patterns may provide excellent targets for further research into their roles and possible use in plant genetic improvement. For example, *GmGLK38*, *GmGLK76*, and *GmGLK79* are explicitly expressed in leaves, while their orthologous genes in Arabidopsis play important roles in leaf development chloroplast formation, and anthocyanin biosynthesis (Fitter et al., 2002; Yasumura et al., 2005).

Protein-protein interaction analysis revealed that *GmGLK* genes encoding proteins play important role in chlorophyll biosynthesis, circadian rhythms, and regulation of flowering (**Figure 8**). In the current study, various *GmGLK* genes interacted with important chlorophyll biosynthesis genes, including three genes such as *GmGLK38*, *GmGLK76*, and *GmGLK79*, which were very strongly connected, as previously reported that these chlorophyll biosynthesis genes play a significant role in chloroplast development in different species (Nakamura et al., 2009; Kobayashi et al., 2013; Sakuraba et al., 2017; Shi et al., 2017; Li M. et al., 2021). Furthermore, four *GmGLKs*, such as *GmGLK13*, *GmGLK70* (*GmLUXc*), *GmGLK74* (*GmLUXb*), and *GmGLK120* encode proteins that strongly interacted with the circadian clock and flowering-related proteins. However, previous studies also supported these connections that the *GmLUXb* and *GmLUXc* form protein complexes with *GmELF3a* and *GmELF4b* and share many different phenotypes, including a circadian oscillator, hypocotyl growth, and flowering regulation in various plants species (Hicks et al., 1996; Liu et al., 2001; Hazen et al., 2005; Onai and Ishiura, 2005; Nozue et al., 2007; Thines and Harmon, 2010; Nusinow et al., 2011).

Excessive concentration of metal ions is hazardous to plant cells. Cu and Cd are two of the most hazardous metal ions. The effects of Cd toxicity on plant biological processes include altered root development, disrupted of regulatory systems, oxidative stress, hampered nutrient uptake, membrane destruction, and, cell death in extreme cases (Song et al., 2017; Chang et al., 2019). The Cu ions level significantly affects several metabolic processes linked to plant development. Both the excess and shortage of Cu can affect vital metabolic processes *in vivo*, comprising regulating plant growth and development (Adrees et al., 2015). Previously, it was reported that Myb-like TF has a significant role in heavy metal toxicity. In Arabidopsis, a loss of function MYB72 mutant showed enhanced metal sensitivity (Van De Mortel et al., 2008). In addition, mutations in OsMYB45 cause a Cd hypersensitive phenotype (Hu et al., 2017). Many MYB-like genes, including MYB4, MYB28, MYB43, MYB48, MYB72, and MYB124, have previously been found to be highly expressed in Arabidopsis under Cd and other metal ion stresses (Van De Mortel et al., 2008). In current study, six *GmGLK* genes under Cd stress and four *GmGLK* genes under Cu stress were highly expressed, including *GmGLK1* (orthologous to Arabidopsis *AtGLK52*), *GmGLK5* (orthologous to Arabidopsis *AtGLK21*), and *GmGLK67* (orthologous to Arabidopsis *AtGLK51*) showed similar expression in both species under Cd and Cu stress treatments (Alam et al., 2022). The differential gene expression and activity of GLKs in Cd and Cu stress provides evidence that GLKs have a potential role during metal ion stress.

## Conclusions

In this study, we discovered 130 *GmGLK* genes encoding TFs in soybean, which were unevenly distributed across 20 chromosomes. The *GmGLK* members were further evenly divided into the five major groups and were separated into clades A, B, C, D, and E of a phylogenetic tree. The gene structure and conserved motifs of *GmGLK* members from the same group or clade share common features, suggesting that they have similar biological activities. According to gene duplication analyses, segmental duplications play a significant role in the expansion of the *GmGLK* family and generation of novel *GmGLK* genes. Data from cis-regulatory element analyses, transcriptomic expression analysis and qRT-PCR experiments in response to cadmium and copper stress treatments revealed that *GmGLK* genes might be involved in soybean expansion and abiotic stress responses. Co-expression analysis identified many crucial *GmGLK* genes strongly connected with chlorophyll biosynthesis, circadian rhythms, and flowering regulatory networks and has provided valuable information for further functional characterization of each *GmGLK* gene across the legume species. In conclusion, our findings have laid the foundation for further functional studies of *GmGLK* genes, which may increase the present knowledge on soybean genetic improvement in response to Cd and Cu stress.

## Data availability statement

The original contributions presented in the study are included in the article/**Supplementary Material**. Further inquiries can be directed to the corresponding author.

## Author contributions

Conceptualization, IA, and LG; methodology, IA, HM, HZ, and QY; software, IA; experiments IA; formal analysis, IA; validation, IA, and LG; investigation, IA; data curation, IA; resources, LG; writing—original draft preparation, IA; writing—review and editing, IA, HM, HZ, QY, and LG; visualization, LG; supervision, LG; project administration, LG; funding acquisition, LG. The manuscript was read and approved for publication by all authors.

## Funding

This work was supported by the Key-Areas Research and Development Program of Guangdong Province (2020B020220008), and the start-up fund from SCAU (to LG).

## Acknowledgments

The authors thank the lab member, the editors, and the reviewers for their useful suggestions.

## Conflict of interest

The authors declare that the research was conducted in the absence of any commercial or financial relationships that could be construed as a potential conflict of interest.

The reviewer AA declared a shared affiliation with the authors to the handling editor at the time of the review.

## Publisher’s note

All claims expressed in this article are solely those of the authors and do not necessarily represent those of their affiliated organizations, or those of the publisher, the editors and the reviewers. Any product that may be evaluated in this article, or claim that may be made by its manufacturer, is not guaranteed or endorsed by the publisher.

## Supplementary material

Supplementary Figure 1 Tomtom analysis of transcription factors for 15 conserved motifs.

Supplementary Table 1 The list and detailed information of soybean GLK members.

Supplementary Table 2 Distribution of soybean GLK members classification based on their additional domain(s).

Supplementary Table 3 GO analysis of soybean GLK members.

Supplementary Table 4 The Ka, Ks, and Ka/Ks analysis and divergence event of duplicated GLK gene pairs in soybean.

Supplementary Table 5 Cis-acting element analysis of soybean GLK member promoter region.

Supplementary Table 6 Sequences of Soybean *GLK* gene primers for qRT-PCR.

## References

[B1] Adrees M. Ali S. Rizwan M. Ibrahim M. Abbas F. Farid M.. (2015). The effect of excess copper on growth and physiology of important food crops: a review. Environ. Sci. pollut. Res. 22, 8148–8162. doi: 10.1007/s11356-015-4496-5 25874438

[B2] Ahmad R. Liu Y. Wang T.-J. Meng Q. Yin H. Wang X.. (2019). GOLDEN2-LIKE transcription factors regulate WRKY40 expression in response to abscisic acid. Plant Physiol. 179, 1844–1860. doi: 10.1104/pp.18.01466 30723180 PMC6446771

[B3] Alam I. Wu X. Yu Q. Ge L. (2022). Comprehensive genomic analysis of G2-like transcription factor genes and their role in development and abiotic stresses in arabidopsis. Diversity 14, 228. doi: 10.3390/d14030228

[B4] Bailey T. L. Elkan C. (1994). Fitting a mixture model by expectation maximization to discover motifs in bipolymers. Proc. Int. Conf. Intell. Syst. Mol. Biol. 2, 28–36. 7584402

[B5] Bari R. Datt Pant B. Stitt M. Scheible W.-R. (2006). PHO2, microRNA399, and PHR1 define a phosphate-signaling pathway in plants. Plant Physiol. 141, 988–999. doi: 10.1104/pp.106.079707 16679424 PMC1489890

[B6] Bhutia K. L. Nongbri E. L. Gympad E. Rai M. Tyagi W. (2020). In silico characterization, and expression analysis of rice golden 2-like (OsGLK) members in response to low phosphorous. Mol. Biol. Rep. 47, 2529–2549. doi: 10.1007/s11033-020-05337-2 32086721

[B7] Birchler J. A. Yang H. (2022). The multiple fates of gene duplications: Deletion, hypofunctionalization, subfunctionalization, neofunctionalization, dosage balance constraints, and neutral variation. Plant Cell. 34, 2466–2474. doi: 10.1093/plcell/koac076 35253876 PMC9252495

[B8] Bohnert H. J. Gong Q. Li P. Ma S. (2006). Unraveling abiotic stress tolerance mechanisms–getting genomics going. Curr. Opin. Plant Biol. 9, 180–188. doi: 10.1016/j.pbi.2006.01.003 16458043

[B9] Caggiano M. P. Yu X. Bhatia N. Larsson A. Ram H. Ohno C. K.. (2017). Cell type boundaries organize plant development. Elife 6, e27421. doi: 10.7554/eLife.27421 28895530 PMC5617630

[B10] Cai R. (2011). Biochemical responses of lemna minor experimentally exposed to cadmium and zinc. Ecotoxicology 20, 815–826. doi: 10.1007/s10646-011-0633-1 21416111

[B11] Cannon S. B. Mitra A. Baumgarten A. Young N. D. May G. (2004). The roles of segmental and tandem gene duplication in the evolution of large gene families in *Arabidopsis thaliana* . BMC Plant Biol. 4, 1–21. doi: 10.1186/1471-2229-4-10 15171794 PMC446195

[B12] Chang C. Yin R. Zhang H. Yao L. (2019). Bioaccumulation and health risk assessment of heavy metals in the soil–rice system in a typical seleniferous area in central China. Environ. Toxicol. Chem. 38, 1577–1584. doi: 10.1002/etc.4443 30994945

[B13] Chen C. Chen H. Zhang Y. Thomas H. R. Frank M. H. He Y.. (2020). TBtools: an integrative toolkit developed for interactive analyses of big biological data. Mol. Plant 13, 1194–1202. doi: 10.1016/j.molp.2020.06.009 32585190

[B14] Chen M. Ji M. Wen B. Liu L. Li S. Chen X.. (2016). GOLDEN 2-LIKE transcription factors of plants. Front. Plant Sci. 7. doi: 10.3389/fpls.2016.01509 PMC5048441 27757121

[B15] Davidson E. H. Jacobs H. T. Britten R. J. (1983). Eukaryotic gene expression: Very short repeats and coordinate induction of genes. Nature 301, 468–470. doi: 10.1038/301468a0 6823326

[B16] Fitter D. W. Martin D. J. Copley M. J. Scotland R. W. Langdale J. A. (2002). GLK gene pairs regulate chloroplast development in diverse plant species. Plant J. 31, 713–727. doi: 10.1046/j.1365-313X.2002.01390.x 12220263

[B17] Garapati P. Xue G.-P. Munné-Bosch S. Balazadeh S. (2015). Transcription factor ATAF1 in arabidopsis promotes senescence by direct regulation of key chloroplast maintenance and senescence transcriptional cascades. Plant Physiol. 168, 1122–1139. doi: 10.1104/pp.15.00567 25953103 PMC4741325

[B18] Garg N. Singla P. (2011). Arsenic toxicity in crop plants: physiological effects and tolerance mechanisms. Environ. Chem. Lett. 9, 303–321. doi: 10.1007/s10311-011-0313-7

[B19] Goldman N. Yang Z. (1994). A codon-based model of nucleotide substitution for protein-coding DNA sequences. Mol. Biol. Evol. 11, 725–736. doi: 10.1093/oxfordjournals.molbev.a040153 7968486

[B20] Gupta S. Stamatoyannopoulos J. A. Bailey T. L. Noble W. S. (2007). Quantifying similarity between motifs. Genome Biol. 8, R24. doi: 10.1186/gb-2007-8-2-r24 17324271 PMC1852410

[B21] Hall L. N. Rossini L. Cribb L. Langdale J. A. (1998). GOLDEN 2: a novel transcriptional regulator of cellular differentiation in the maize leaf. Plant Cell. 10, 925–936. doi: 10.1105/tpc.10.6.925 9634581 PMC144032

[B22] Han X.-Y. Li P.-X. Zou L.-J. Tan W.-R. Zheng T. Zhang D.-W.. (2016). GOLDEN2-LIKE transcription factors coordinate the tolerance to cucumber mosaic virus in arabidopsis. Biochem. Biophys. Res. Commun. 477, 626–632. doi: 10.1016/j.bbrc.2016.06.110 27346129

[B23] Hazen S. P. Schultz T. F. Pruneda-Paz J. L. Borevitz J. O. Ecker J. R. Kay S. A. (2005). LUX ARRHYTHMO encodes a myb domain protein essential for circadian rhythms. PNAS 102, 10387–10392. doi: 10.1073/pnas.0503029102 16006522 PMC1177380

[B24] Hicks K. A. Millar A. J. Carré I. A. Somers D. E. Straume M. Meeks-Wagner D. R.. (1996). Conditional circadian dysfunction of the arabidopsis early-flowering 3 mutant. Science 274, 790–792. doi: 10.1126/science.274.5288.790 8864121

[B25] Hu S. Yu Y. Chen Q. Mu G. Shen Z. Zheng L. (2017). OsMYB45 plays an important role in rice resistance to cadmium stress. Plant Sci. 264, 1–8. doi: 10.1016/j.plantsci.2017.08.002 28969789

[B26] Jalmi S. K. Bhagat P. K. Verma D. Noryang S. Tayyeba S. Singh K.. (2018). Traversing the links between heavy metal stress and plant signaling. Front. Plant Sci. 9. doi: 10.3389/fpls.2018.00012 PMC5807407 29459874

[B27] Jones D. T. Taylor W. R. Thornton J. M. (1992). The rapid generation of mutation data matrices from protein sequences. Bioinformatics 8, 275–282. doi: 10.1093/bioinformatics/8.3.275 1633570

[B28] Junfang L. Jia Z. He L. Tingting Z. Jingfu L. (2017). Research progress of plant GOLDEN2-like transcription factor. Mol. Plant Breed. 10, 3949–3956. doi: 10.3390/d14030228

[B29] Kakizaki T. Matsumura H. Nakayama K. Che F.-S. Terauchi R. Inaba T. (2009). Coordination of plastid protein import and nuclear gene expression by plastid-to-nucleus retrograde signaling. Plant Physiol. 151, 1339–1353. doi: 10.1104/pp.109.145987 19726569 PMC2773054

[B30] Kiba T. Inaba J. Kudo T. Ueda N. Konishi M. Mitsuda N.. (2018). Repression of nitrogen starvation responses by members of the arabidopsis GARP-type transcription factor NIGT1/HRS1 subfamily. Plant Cell. 30, 925–945. doi: 10.1105/tpc.17.00810 29622567 PMC5969275

[B31] Kim E. Hwang S. Lee I. (2017). SoyNet: a database of co-functional networks for soybean *Glycine max* . Nucleic Acids Res. 45, D1082–d1089. doi: 10.1093/nar/gkw704 27492285 PMC5210602

[B32] Kobayashi K. Sasaki D. Noguchi K. Fujinuma D. Komatsu H. Kobayashi M.. (2013). Photosynthesis of root chloroplasts developed in arabidopsis lines overexpressing GOLDEN2-LIKE transcription factors. Plant Cell Physiol. 54, 1365–1377. doi: 10.1093/pcp/pct086 23749810 PMC3730084

[B33] Kumar S. Stecher G. Li M. Knyaz C. Tamura K. (2018). MEGA X: molecular evolutionary genetics analysis across computing platforms. Mol. Biol. Evol. 35, 1547. doi: 10.1093/molbev/msy096 29722887 PMC5967553

[B34] Leister D. Kleine T. (2016). Definition of a core module for the nuclear retrograde response to altered organellar gene expression identifies GLK overexpressors as gun mutants. Physiol. Plant 157, 297–309. doi: 10.1111/ppl.12431 26876646

[B35] Lescot M. Déhais P. Thijs G. Marchal K. Moreau Y. Van De Peer Y.. (2002). PlantCARE, a database of plant cis-acting regulatory elements and a portal to tools for in silico analysis of promoter sequences. Nucleic Acids Res. 30, 325–327. doi: 10.1093/nar/30.1.325 11752327 PMC99092

[B36] Liew L. C. Singh M. B. Bhalla P. L. (2017). A novel role of the soybean clock gene LUX ARRHYTHMO in male reproductive development. Sci. Rep. 7, 10605. doi: 10.1038/s41598-017-10823-y 28878247 PMC5587693

[B37] Li M. Lee K. P. Liu T. Dogra V. Duan J. Li M.. (2021). Antagonistic modules regulate photosynthesis-associated nuclear genes *via* GOLDEN2-LIKE transcription factors. Plant Physiol. 188, 2308–2324. doi: 10.1093/plphys/kiab600 PMC8968271 34951648

[B38] Li X. Mao X. Xu Y. Li Y. Zhao N. Yao J.. (2021). Comparative transcriptomic analysis reveals the coordinated mechanisms of populus× canadensis ‘Neva’leaves in response to cadmium stress. Ecotox. Environ. Safe. 216, 112179. doi: 10.1016/j.ecoenv.2021.112179 33798869

[B39] Liu X. L. Covington M. F. Fankhauser C. Chory J. Wagner D. R. (2001). ELF3 encodes a circadian clock–regulated nuclear protein that functions in an arabidopsis PHYB signal transduction pathway. Plant Cell. 13, 1293–1304. doi: 10.1105/TPC.000475 11402161 PMC135570

[B40] Liu J. Mehari T. Xu Y. Umer M. Hou Y. Wang Y.. (2021b). GhGLK1 a key candidate gene from GARP family enhances cold and drought stress tolerance in cotton. Front. Plant Sci. 12. doi: 10.3389/fpls.2021.759312 PMC8725998 34992618

[B41] Liu F. Xu Y. Han G. Zhou L. Ali A. Zhu S.. (2016). Molecular evolution and genetic variation of G2-like transcription factor genes in maize. PloS One 11, e0161763. doi: 10.1371/journal.pone.0161763 27560803 PMC4999087

[B42] Liu D. Zhao D. Li X. Zeng Y. (2021a). AtGLK2, an arabidopsis GOLDEN2-LIKE transcription factor, positively regulates anthocyanin biosynthesis *via* AtHY5-mediated light signaling. Plant Growth Regul. 96, 79–90. doi: 10.1007/s10725-021-00759-9

[B43] Li X. Wang P. Li J. Wei S. Yan Y. Yang J.. (2020). Maize GOLDEN2-LIKE genes enhance biomass and grain yields in rice by improving photosynthesis and reducing photoinhibition. Commun. Biol. 3, 1–12. doi: 10.1038/s42003-020-0887-3 32238902 PMC7113295

[B44] Ludewig U. Vatov E. Hedderich D. Neuhäuser B. (2021). Adjusting plant nutrient acquisition to fluctuating availability: transcriptional co-regulation of the nitrate and phosphate deprivation responses in roots. J. Exp. Bot. 72, 3500–3503. doi: 10.1093/jxb/erab131 33948653 PMC8096598

[B45] Maeda Y. Konishi M. Kiba T. Sakuraba Y. Sawaki N. Kurai T.. (2018). A NIGT1-centred transcriptional cascade regulates nitrate signalling and incorporates phosphorus starvation signals in arabidopsis. Nat. Commun. 9, 1–14. doi: 10.1038/s41467-018-03832-6 29636481 PMC5893545

[B46] Mustafa G. Komatsu S. (2016). Toxicity of heavy metals and metal-containing nanoparticles on plants. Biochim. Biophys. Acta Proteins Proteom. . 1864, 932–944. doi: 10.1016/j.bbapap.2016.02.020 26940747

[B47] Nagatoshi Y. Mitsuda N. Hayashi M. Inoue S.-I. Okuma E. Kubo A.. (2016). GOLDEN 2-LIKE transcription factors for chloroplast development affect ozone tolerance through the regulation of stomatal movement. PNAS 113, 4218–4223. doi: 10.1073/pnas.1513093113 27035938 PMC4839443

[B48] Nakamura H. Muramatsu M. Hakata M. Ueno O. Nagamura Y. Hirochika H.. (2009). Ectopic overexpression of the transcription factor OsGLK1 induces chloroplast development in non-green rice cells. Plant Cell Physiol. 50, 1933–1949. doi: 10.1093/pcp/pcp138 19808806 PMC2775961

[B49] Nozue K. Covington M. F. Duek P. D. Lorrain S. Fankhauser C. Harmer S. L.. (2007). Rhythmic growth explained by coincidence between internal and external cues. Nature 448, 358–361. doi: 10.1038/nature05946 17589502

[B50] Nusinow D. A. Helfer A. Hamilton E. E. King J. J. Imaizumi T. Schultz T. F.. (2011). The ELF4–ELF3–LUX complex links the circadian clock to diurnal control of hypocotyl growth. Nature 475, 398–402. doi: 10.1038/nature10182 21753751 PMC3155984

[B51] Onai K. Ishiura M. (2005). PHYTOCLOCK 1 encoding a novel GARP protein essential for the arabidopsis circadian clock. Genes Cells 10, 963–972. doi: 10.1111/j.1365-2443.2005.00892.x 16164597

[B52] Preuss S. B. Meister R. Xu Q. Urwin C. P. Tripodi F. A. Screen S. E.. (2012). Expression of the *Arabidopsis thaliana* BBX32 gene in soybean increases grain yield. PloS One 7, e30717. doi: 10.1371/journal.pone.0030717 22363475 PMC3281879

[B53] Protparam E. (2017). ExPASy-ProtParam tool (Lausanne, Switzerland: SIB).

[B54] Qin M. Zhang B. Gu G. Yuan J. Yang X. Yang J.. (2021). Genome-wide analysis of the G2-like transcription factor genes and their expression in different senescence stages of tobacco (*Nicotiana tabacum* l.). Front. Genet. 12. doi: 10.3389/fgene.2021.626352 PMC8202009 34135936

[B55] Ram H. Sahadevan S. Gale N. Caggiano M. P. Yu X. Ohno C.. (2020). An integrated analysis of cell-type specific gene expression reveals genes regulated by REVOLUTA and KANADI1 in the arabidopsis shoot apical meristem. PloS Genet. 16, e1008661. doi: 10.1371/journal.pgen.1008661 32294082 PMC7266345

[B56] Ramsay N. A. Glover B. J. (2005). MYB–bHLH–WD40 protein complex and the evolution of cellular diversity. Trends Plant Sci. 10, 63–70. doi: 10.1016/j.tplants.2004.12.011 15708343

[B57] Rauf M. Arif M. Dortay H. Matallana-Ramírez L. P. Waters M. T. Gil Nam H.. (2013). ORE1 balances leaf senescence against maintenance by antagonizing G2-like-mediated transcription. EMBO Rep. 14, 382–388. doi: 10.1038/embor.2013.24 23459204 PMC3615665

[B58] Riechmann J. L. Heard J. Martin G. Reuber L. Jiang C.-Z. Keddie J.. (2000). Arabidopsis transcription factors: genome-wide comparative analysis among eukaryotes. science 290, 2105–2110. doi: 10.1126/science.290.5499.210 11118137

[B59] Rossini L. Cribb L. Martin D. J. Langdale J. A. (2001). The maize golden2 gene defines a novel class of transcriptional regulators in plants. Plant Cell 13, 1231–1244. doi: 10.1105/tpc.13.5.1231 11340194 PMC135554

[B60] Sakuraba Y. Kim E.-Y. Han S.-H. Piao W. An G. Todaka D.. (2017). Rice phytochrome-interacting factor-Like1 (OsPIL1) is involved in the promotion of chlorophyll biosynthesis through feed-forward regulatory loops. J. Exp. Bot. 68, 4103–4114. doi: 10.1093/jxb/erx231 28922754 PMC5853433

[B61] Salazar M. J. Rodriguez J. H. Nieto G. L. Pignata M. L. (2012). Effects of heavy metal concentrations (Cd, zn and Pb) in agricultural soils near different emission sources on quality, accumulation and food safety in soybean [*Glycine max* (L.) Merrill]. J. Hazard. Mater. 233, 244–253. doi: 10.1016/j.jhazmat.2012.07.026 22835772

[B62] Savitch L. V. Subramaniam R. Allard G. C. Singh J. (2007). The GLK1 ‘regulon’encodes disease defense related proteins and confers resistance to fusarium graminearum in arabidopsis. Biochem. Biophys. Res. Commun. 359, 234–238. doi: 10.1016/j.bbrc.2007.05.084 17533111

[B63] Schmutz J. Cannon S. B. Schlueter J. Ma J. Mitros T. Nelson W.. (2010). Genome sequence of the palaeopolyploid soybean. nature 463, 178–183. doi: 10.1038/nature08670 20075913

[B64] Schreiber K. J. Nasmith C. G. Allard G. Singh J. Subramaniam R. Desveaux D. (2011). Found in translation: high-throughput chemical screening in *Arabidopsis thaliana* identifies small molecules that reduce fusarium head blight disease in wheat. Mol. Plant Microbe Interact. 24, 640–648. doi: 10.1094/MPMI-09-10-0210 21303209

[B65] Sharma S. S. Dietz K.-J. (2006). The significance of amino acids and amino acid-derived molecules in plant responses and adaptation to heavy metal stress. J. Exp. Bot. 57, 711–726. doi: 10.1093/jxb/erj073 16473893

[B66] Shi K. Gu J. Guo H. Zhao L. Xie Y. Xiong H.. (2017). Transcriptome and proteomic analyses reveal multiple differences associated with chloroplast development in the spaceflight-induced wheat albino mutant mta. PloS One 12, e0177992. doi: 10.1371/journal.pone.0177992 28542341 PMC5443577

[B67] Shin D. H. Choi M.-G. Kang C.-S. Park C.-S. Choi S.-B. Park Y.-I. (2016). A wheat R2R3-MYB protein PURPLE PLANT1 (TaPL1) functions as a positive regulator of anthocyanin biosynthesis. Biochem. Biophys. Res. Commun. 469, 686–691. doi: 10.1016/j.bbrc.2015.12.001 26692488

[B68] Sievers F. Higgins D. G. (2014). “Clustal omega, accurate alignment of very large numbers of sequences,” in Multiple sequence alignment methods, vol. 1. (Totowa, NJ, Springer: Humana Press), 105–116. doi: 10.1007/978-1-62703-646-7_6 24170397

[B69] Song Y. Jin L. Wang X. (2017). Cadmium absorption and transportation pathways in plants. Int. J. Phytoremediation. 19, 133–141. doi: 10.1080/15226514.2016.1207598 27409403

[B70] Sun L. Song L. Zhang Y. Zheng Z. Liu D. (2016). Arabidopsis PHL2 and PHR1 act redundantly as the key components of the central regulatory system controlling transcriptional responses to phosphate starvation. Plant Physiol. 170, 499–514. doi: 10.1104/pp.15.01336 26586833 PMC4704584

[B71] Suo J. Liang X. Pu L. Zhang Y. Xue Y. (2003). Identification of GhMYB109 encoding a R2R3 MYB transcription factor that expressed specifically in fiber initials and elongating fibers of cotton (Gossypium hirsutum l.). Biochim. Biophys. Acta Gene Regul. Mech. BBA-GENE. Regul. Mech. 1630, 25–34. doi: 10.1016/j.bbaexp.2003.08.009 14580676

[B72] Suyama M. Torrents D. Bork P. (2006). PAL2NAL: robust conversion of protein sequence alignments into the corresponding codon alignments. Nucleic Acids Res. 34, W609–W612. doi: 10.1093/nar/gkl315 16845082 PMC1538804

[B73] Szklarczyk D. Gable A. L. Lyon D. Junge A. Wyder S. Huerta-Cepas J.. (2018). STRING v11: protein–protein association networks with increased coverage, supporting functional discovery in genome-wide experimental datasets. Nucleic Acids Res. 47, D607–D613. doi: 10.1093/nar/gky1131 PMC6323986 30476243

[B74] Taketa S. Hattori M. Takami T. Himi E. Sakamoto W. (2021). Mutations in a Golden2-like gene cause reduced seed weight in barley albino lemma 1 mutants. Plant Cell Physiol 62, 447–457. doi: 10.1093/pcp/pcab001 33439257

[B75] Thines B. Harmon F. G. (2010). Ambient temperature response establishes ELF3 as a required component of the core arabidopsis circadian clock. PNAS 107, 3257–3262. doi: 10.1073/pnas.0911006107 20133619 PMC2840299

[B76] Tien N. Sabelis M. Egas M. (2015). Inbreeding depression and purging in a haplodiploid: gender-related effects. Heredity 114, 327–332. doi: 10.1038/hdy.2014.106 25407077 PMC4815584

[B77] Ueda Y. Kiba T. Yanagisawa S. (2020). Nitrate-inducible NIGT1 proteins modulate phosphate uptake and starvation signalling *via* transcriptional regulation of SPX genes. Plant J. 102, 448–466. doi: 10.1111/tpj.14637 31811679

[B78] Uehara T. N. Mizutani Y. Kuwata K. Hirota T. Sato A. Mizoi J.. (2019). Casein kinase 1 family regulates PRR5 and TOC1 in the arabidopsis circadian clock. PNAS 116, 11528–11536. doi: 10.1073/pnas.1903357116 31097584 PMC6561244

[B79] Van De Mortel J. E. Schat H. Moerland P. D. Van Themaat E. V. L. van der Ent S. Blankestijn H.. (2008). Expression differences for genes involved in lignin, glutathione and sulphate metabolism in response to cadmium in arabidopsis thaliana and the related Zn/Cd-hyperaccumulator thlaspi caerulescens. Plant Cell Environ. 31, 301–324. doi: 10.1111/j.1365-3040.2007.01764.x 18088336

[B80] Wa Lwalaba J. L. Zvobgo G. Gai Y. Issaka J. H. Mwamba T. M. Louis L. T.. (2021). Transcriptome analysis reveals the tolerant mechanisms to cobalt and copper in barley. Ecotox. Environ. Safe. 209, 111761. doi: 10.1016/j.ecoenv.2020.111761 33333341

[B81] Wang X. Wang H.-F. Chen Y. Sun M.-M. Wang Y. Chen Y.-F. (2020b). The transcription factor NIGT1. 2 modulates both phosphate uptake and nitrate influx during phosphate starvation in arabidopsis and maize. Plant Cell 32, 3519–3534. doi: 10.1105/tpc.20.00361 32958562 PMC7610294

[B82] Wang S. Wei M. Cheng H. Wu B. Du D. Wang C. (2020a). Indigenous plant species and invasive alien species tend to diverge functionally under heavy metal pollution and drought stress. Ecotoxicol. Environ.Saf. 205, 111160. doi: 10.1016/j.ecoenv.2020.111160 32853864

[B83] Wang Z.-Y. Zhao S. Liu J.-F. Zhao H.-Y. Sun X.-Y. Wu T.-R.. (2022). Genome-wide identification of tomato golden 2-like transcription factors and abiotic stress related members screening. BMC Plant Biol. 22, 1–17. doi: 10.1186/s12870-022-03460-9 35196981 PMC8864820

[B84] Waters M. T. Moylan E. C. Langdale J. A. (2008). GLK transcription factors regulate chloroplast development in a cell-autonomous manner. Plant J. 56, 432–444. doi: 10.1111/j.1365-313X.2008.03616.x 18643989

[B85] Waters M. T. Wang P. Korkaric M. Capper R. G. Saunders N. J. Langdale J. A. (2009). GLK transcription factors coordinate expression of the photosynthetic apparatus in arabidopsis. Plant Cell 21, 1109–1128. doi: 10.1105/tpc.108.065250 19376934 PMC2685620

[B86] Xu Y. Li H.-N. Li G.-J. Wang X. Cheng L.-G. Zhang Y.-M. (2011). Mapping quantitative trait loci for seed size traits in soybean (Glycine max l. merr.). Theor. Appl. Genet. 122, 581–594. doi: 10.1007/s00122-010-1471-x 20981403

[B87] Yang Z. Goldman N. Friday A. (1994). Comparison of models for nucleotide substitution used in maximum-likelihood phylogenetic estimation. Mol. Biol. Evol. 11, 316–324. doi: 10.1093/oxfordjournals.molbev.a040112 8170371

[B88] Yan A. Wang Y. Tan S. N. Mohd Yusof M. L. Ghosh S. Chen Z. (2020). Phytoremediation: a promising approach for revegetation of heavy metal-polluted land. Front. Plant Sci. 11. doi: 10.3389/fpls.2020.00359 PMC7203417 32425957

[B89] Yasumura Y. Moylan E. C. Langdale J. A. (2005). A conserved transcription factor mediates nuclear control of organelle biogenesis in anciently diverged land plants. Plant Cell 17, 1894–1907. doi: 10.1105/tpc.105.033191 15923345 PMC1167540

[B90] Zaikina E. A. Rumyantsev S. D. Sarvarova E. R. Kuluev B. R. (2019). Transcription factor genes involved in plant response to abiotic stress factors. Ecol. Genet. 17, 47–58. doi: 10.17816/ecogen17347-58

[B91] Zhang D. Liu X. Ma J. Yang H. Zhang W. Li C. (2019). Genotypic differences and glutathione metabolism response in wheat exposed to copper. Environ. Exp. Bot. 157, 250–259. doi: 10.1016/j.envexpbot.2018.06.032

[B92] Zhao W. Cheng Y. Zhang C. You Q. Shen X. Guo W.. (2017). Genome-wide identification and characterization of circular RNAs by high throughput sequencing in soybean. Sci. Rep. 7, 1–11. doi: 10.1038/s41598-017-05922-9 28717203 PMC5514102

[B93] Zhao Z. Shuang J. Li Z. Xiao H. Liu Y. Wang T.. (2021). Identification of the golden-2-like transcription factors gene family in gossypium hirsutum. PeerJ 9, e12484. doi: 10.7717/peerj.12484 34820202 PMC8603818

[B94] Zhou J. Jiao F. Wu Z. Li Y. Wang X. He X.. (2008). OsPHR2 is involved in phosphate-starvation signaling and excessive phosphate accumulation in shoots of plants. Plant Physiol. 146, 1673–1686. doi: 10.1104/pp.107.111443 18263782 PMC2287342

[B95] Zhu X. Zhang L. Kuang C. Guo Y. Huang C. Deng L.. (2018). Important photosynthetic contribution of silique wall to seed yield-related traits in arabidopsis thaliana. Photosynth. Res. 137, 493–501. doi: 10.1007/s11120-018-0532-x 29959749

