# Supplementary figures and images for: Identification of GOLDEN2-like transcription factor genes in soybeans and their role in regulating plant development and metal ion stresses
Source: Front Plant Sci. 2022 Nov 11;13:1052659. doi: 10.3389/fpls.2022.1052659 (PMC9691782; doi:10.3389/fpls.2022.1052659)

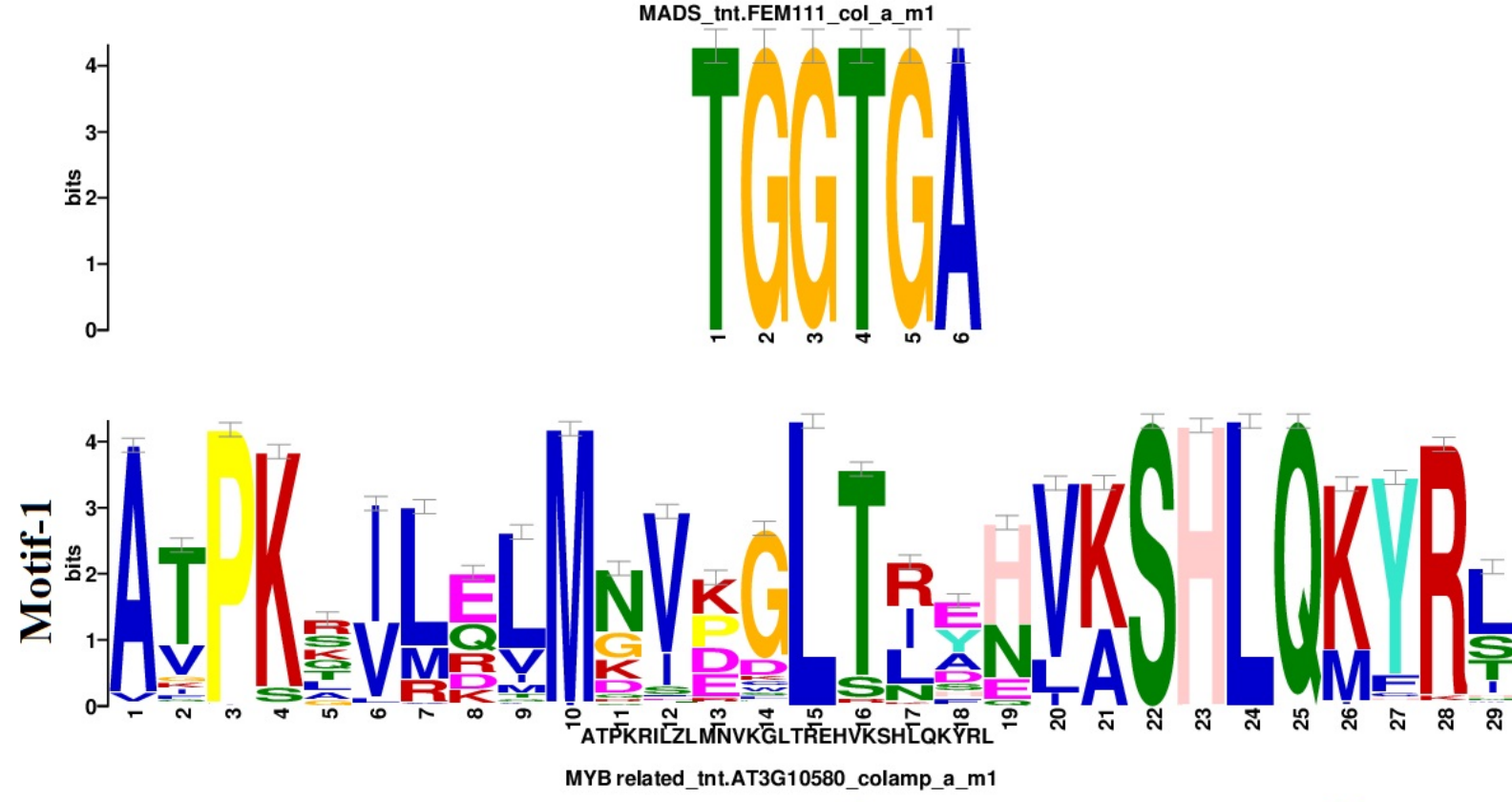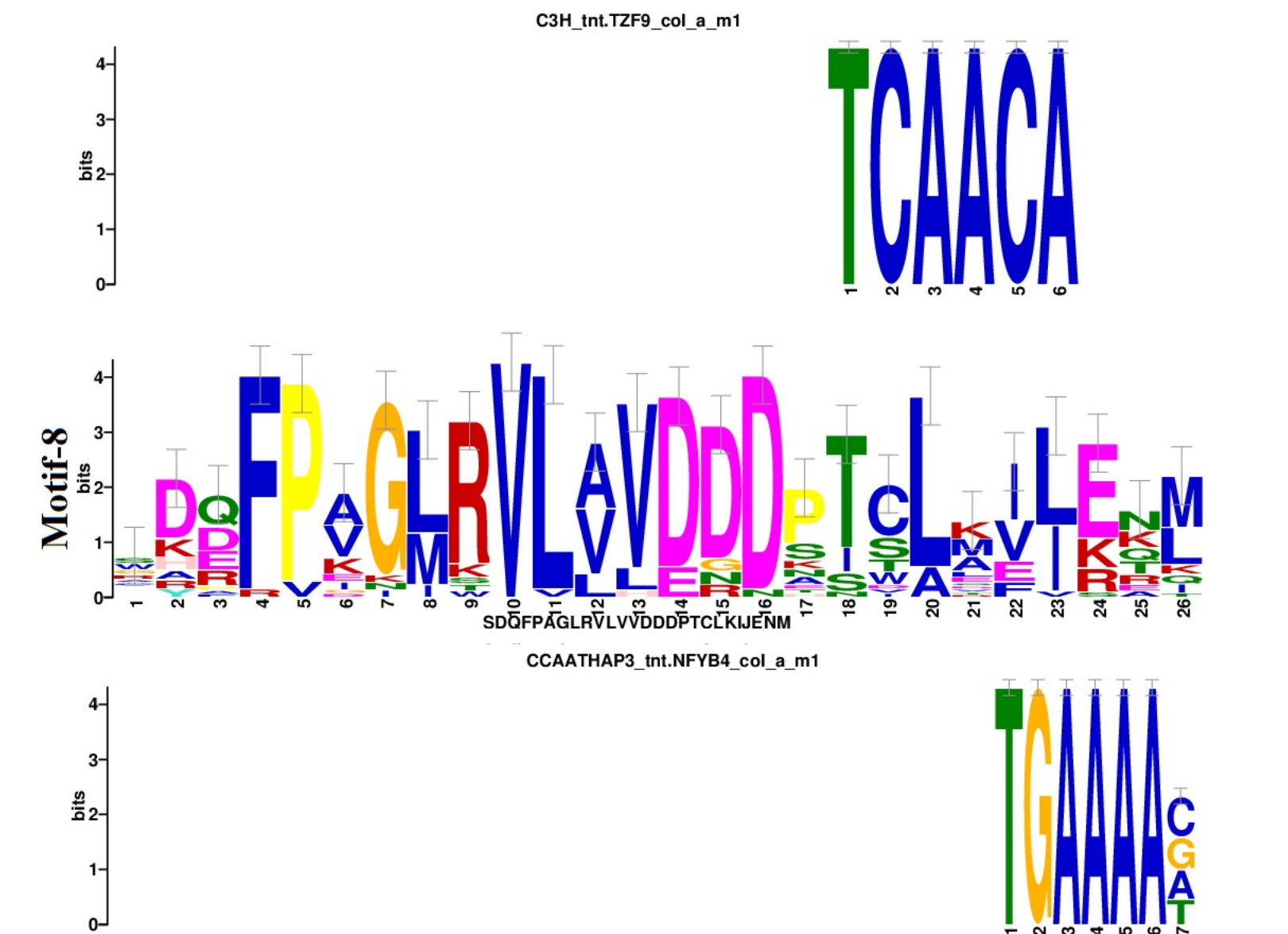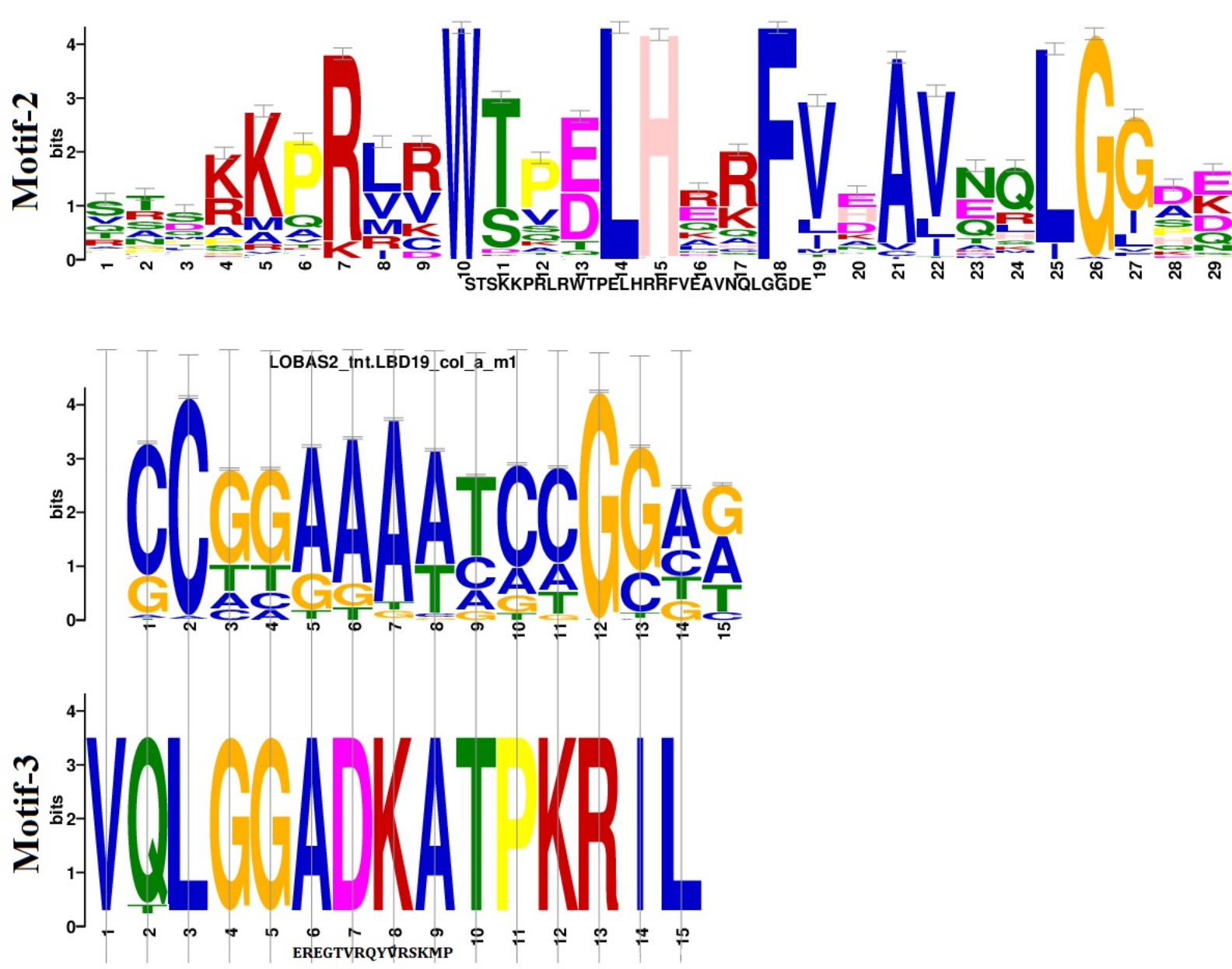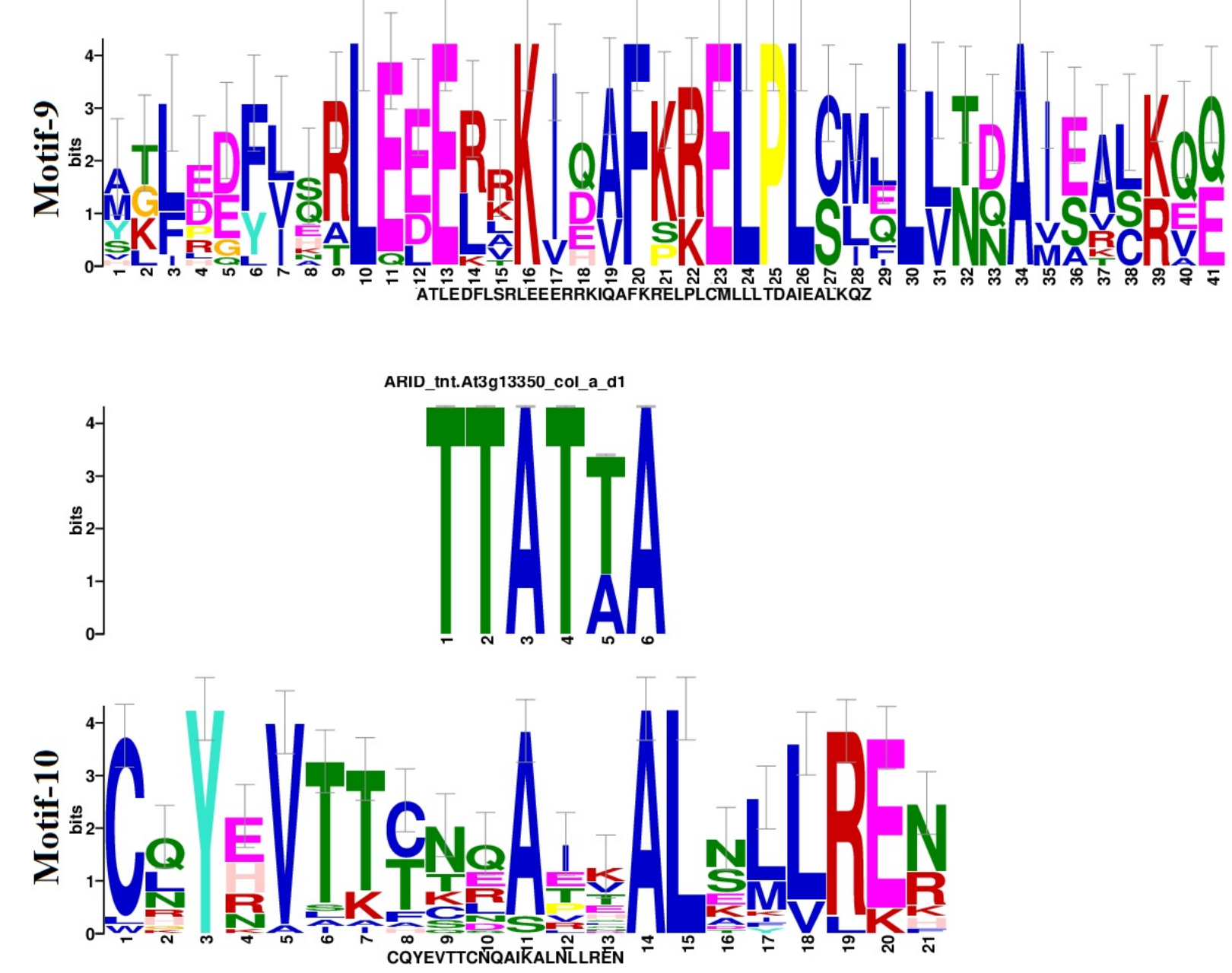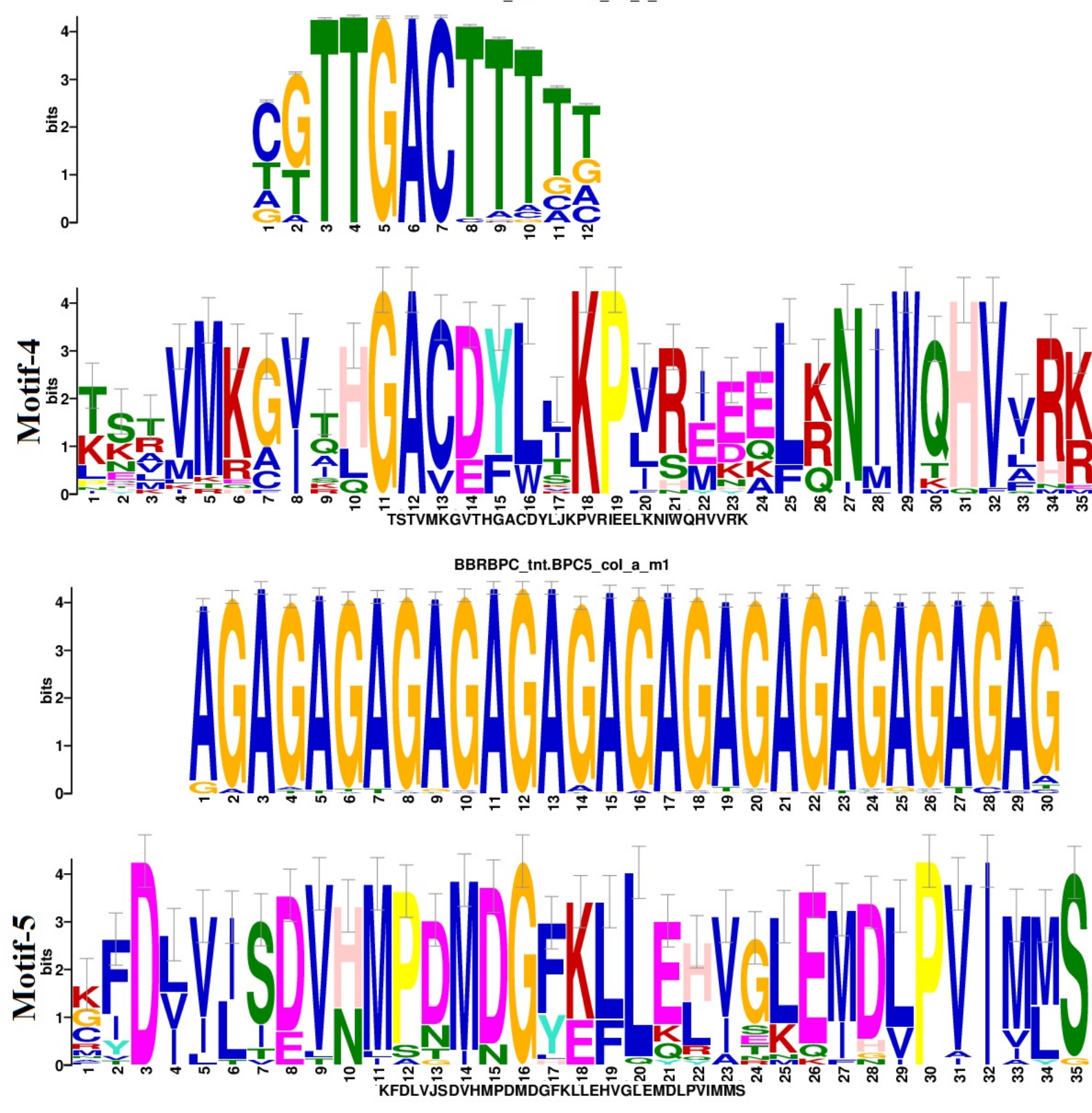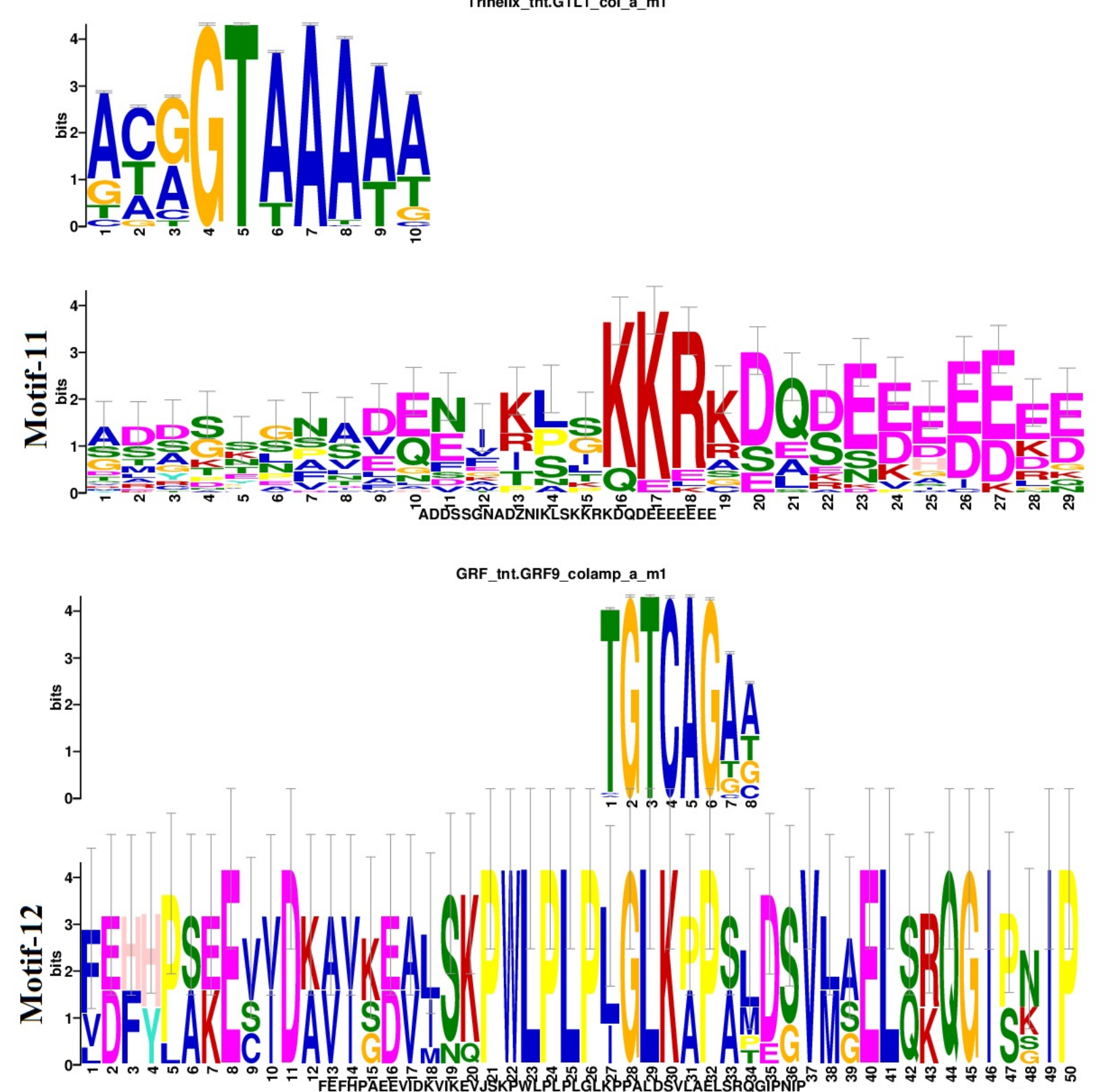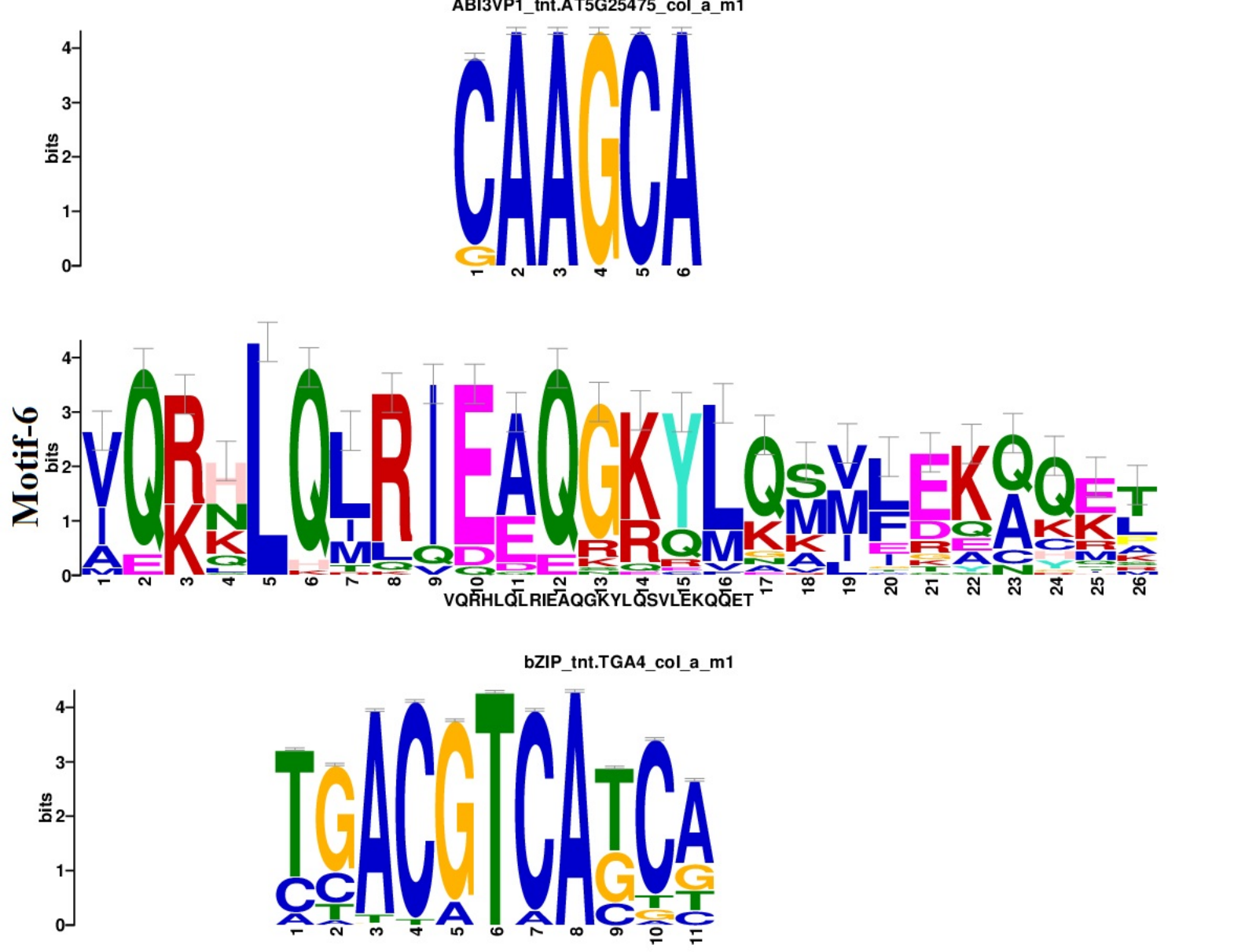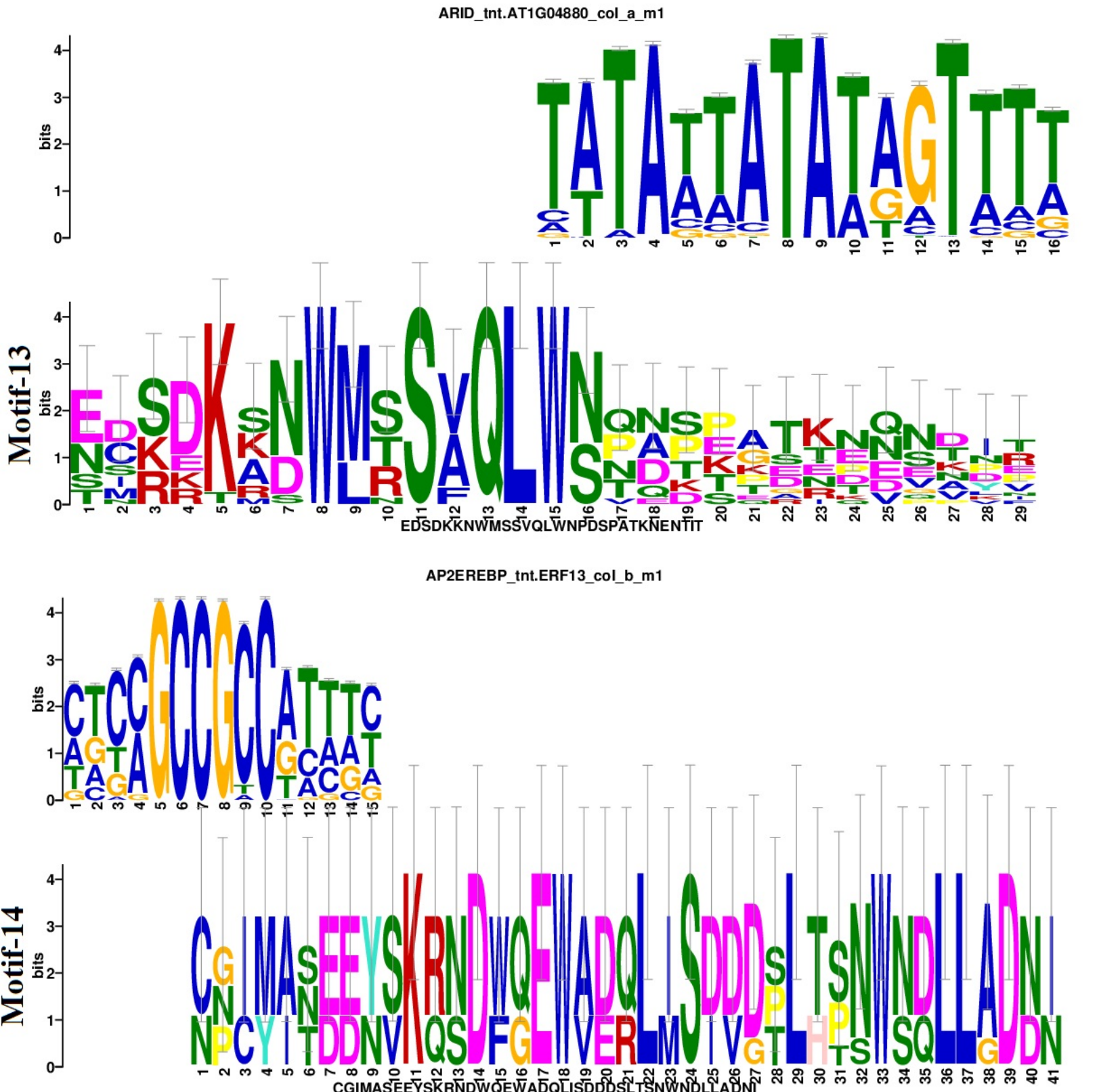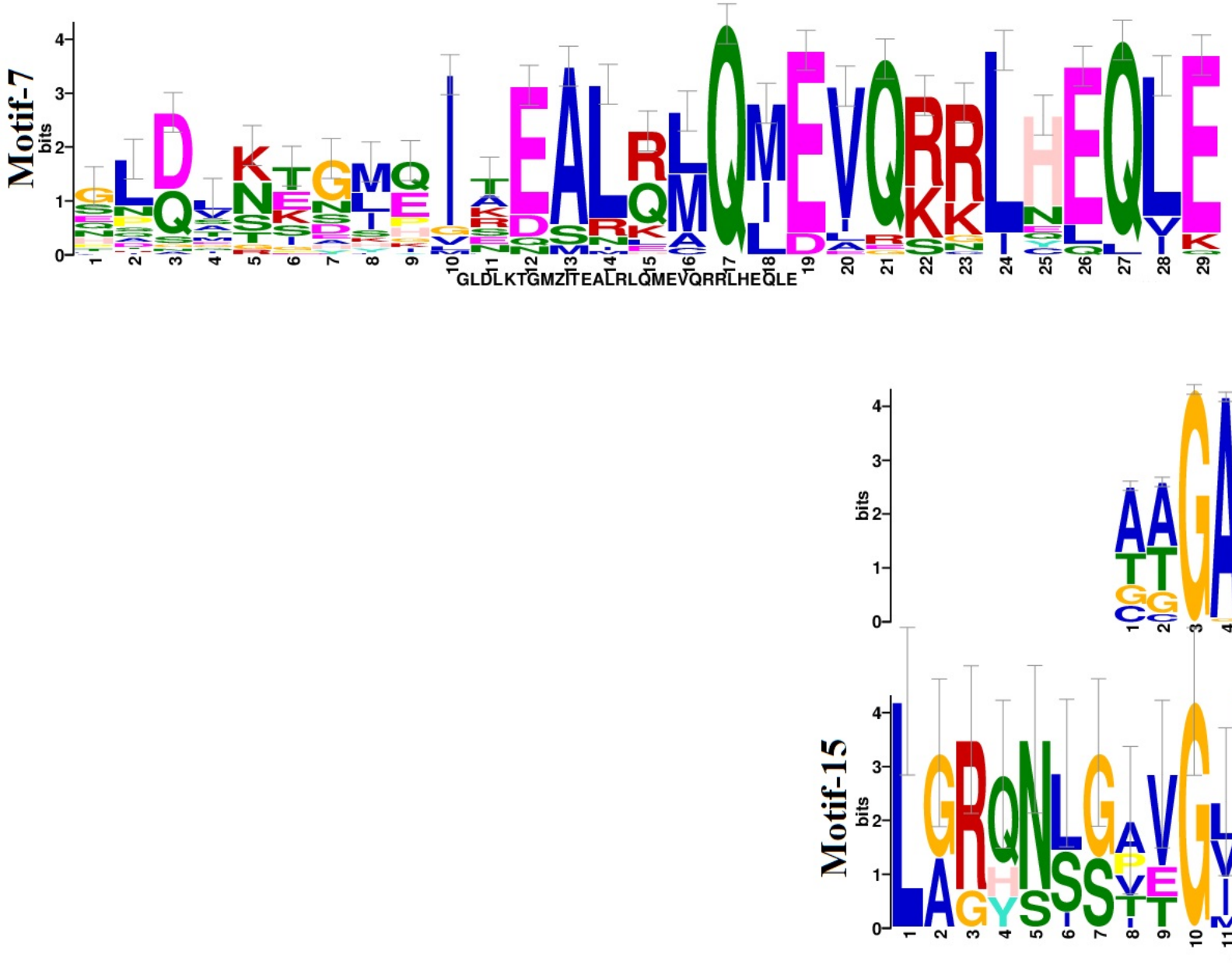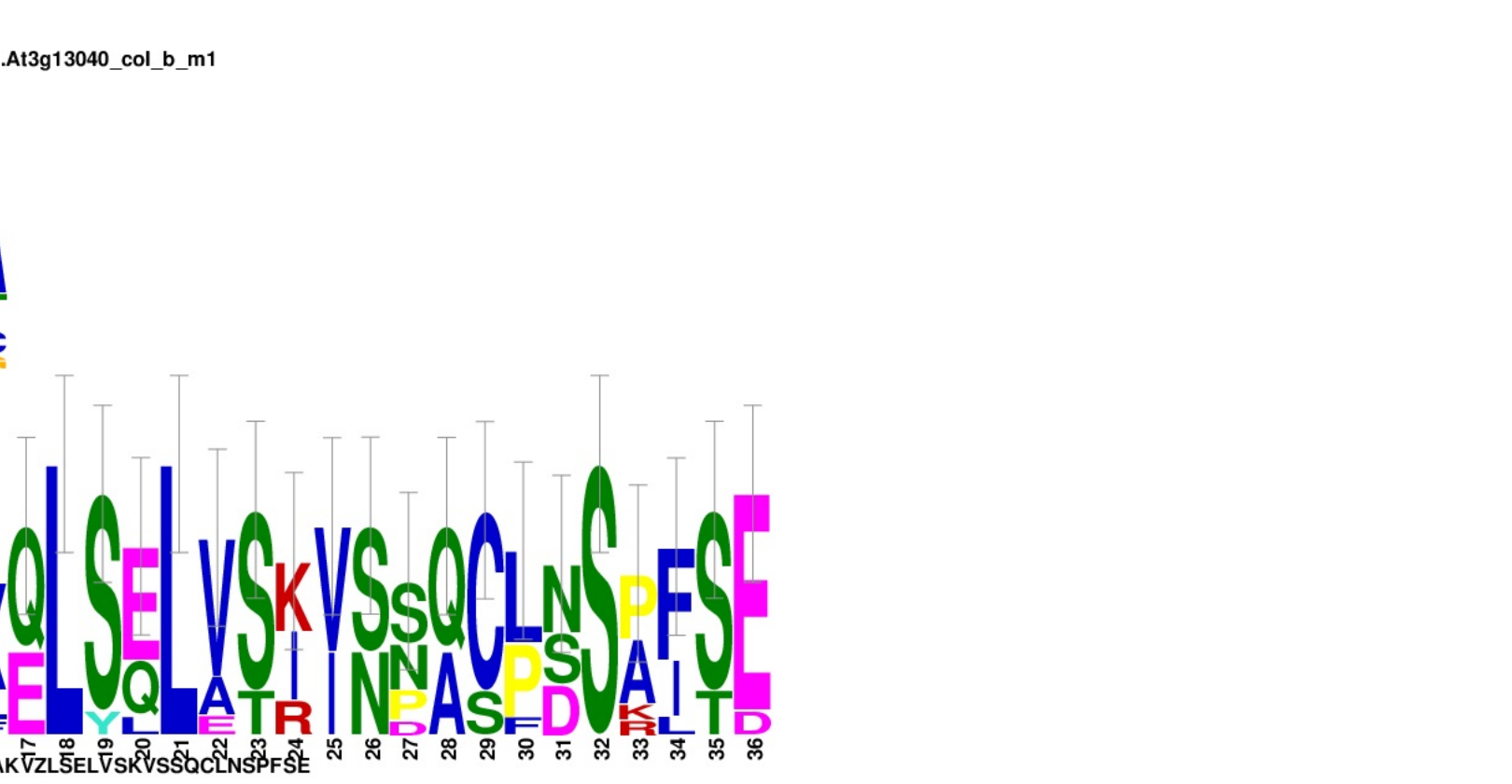

Supplement: Supplementary Figure 1 — Tomtom analysis of transcription factors for 15 conserved motifs. [file DataSheet_1.zip › Supplementary Figure. S1.pdf]
